# Supporting a Healthy Planet, Healthy People and Health Equity through Urban and Territorial Planning

**DOI:** 10.1080/02697459.2021.2000144

**Published:** 2022-01-23

**Authors:** Grant Marcus, José Siri, Franz Gatzweiler, Carlos Dora, Jens Aerts, Sarah Nandudu, Alice Claeson, Pamela Carbajal, Nathalie Roebbel, Laura Petrella, Thiago Hérick de Sá

**Affiliations:** a Environmental Stewardship for Health, UK; b Wellcome Trust, UK; c Urban Health and Wellbeing Programme, International Science Council (ISC), China; d International Society of Urban Health (ISUH), USA; e International Society of City and Regional Planners (Isocarp, The Hague, The Netherlands; f Slum Dwellers International, Cape Town, South Africa; g Department of Emergency Medicine , Vrinnevi Hospital, Norrköping, Sweden; h UN-Habitat, Kenya; i World Health Organization, Geneva

**Keywords:** Healthy urban planning, urban and territorial planning, healthy cities, health equity, planetary health

## Abstract

The form of human settlements impacts on planetary health, population health and health equity. Yet goals for urban and territorial planning are only tangentially linked to public health outcomes. The WHO and UN-Habitat support actions to bring health to the fore in planning and design of human settlements, recently publishing ‘Integrating Health in Urban and Territorial Planning: a sourcebook’ focusing on ‘why’ action is needed, ‘how’ to initiate it; and curating several existing resources on ‘what’ to do. Recommendations for research, policy and practice include calls for rapid development of closer relationships between public health and spatial planning.

## Introduction

The purpose of this paper is to make the case for centering health as an input and outcome of good planning of urban environments, and provide an account of why and how a recent publication from the World Health Organization (WHO) and UN-Habitat ‘Integrating Health in Urban and Territorial Planning: a sourcebook’ (UN-Habitat & WHO 2020), hereafter called the Sourcebook, was developed and what shaped its final form. With the New Urban Agenda as a backdrop, we position this account within the recent history of research that is providing a stronger understanding of how built environments impact health and health equity and the mechanisms needed for change (Prüss-Ustün 2017), Crane *et al*., 2021). The context is also one in which this understanding is leading to a range of policy documents and practice reports that attempt to use built environment form and urban planning to support population health and health equity particularly, though not exclusively, in association to non-communicable disease. The team leading what resulted in this Sourcebook arose from those involved with developing ‘Health is the Pulse of the New Urban Agenda’ (WHO, 2016). At the WHO, these personnel were based at the Department of Environment, Climate Change and Health, Geneva, and at UN-Habitat they were based at the Planning Finance and Economy Section, Urban Practices Branch, Nairobi. To fulfil the promise of ‘Health is the Pulse of the New Urban Agenda’, the two agencies wanted guidelines on urban and territorial planning that would use health as a lens (UN-Habitat & WHO 2018a)). The research, commencing in 2018, was undertaken by Grant under supervision of the two agencies, withpeer review at critical stages by the wider author team.

Communities of interest, whose aim is to support population health through urban spatial policy, have contributed several of the tools and case studies and add another dimension to the discussion on how to integrate health in urban and territorial planning. This is reflected in the development of the Sourcebook and the authorship of this paper, with the engagement of the International Society of City and Regional Planners (ISOCARP), Slum Dwellers International (SDI),the Urban Health and Wellbeing Programme of the International Science Council (ISC), the International Society for Urban Health (ISUH) and the Wellcome Trust.

The paper concludes with the next steps that this community is considering to extend the approach developed and advocated in the Sourcebook, and a call for other partners to be involved.

### Situating the Sourcebook

The Sourcebook closely follows the conventions of International Guidance on Urban and Territorial Planning (UN-Habitat, 2015) in being aimed at actors and stakeholders at all levels who are involved with territorial planning and design, with the addition of public health practitioners as a new group who need to be involved. While containing links to numerous resources on ‘what’ to do, it sets these within a narrative of ‘how’ to integrate health in urban and territorial planning focussing on extending the concept of ‘entry points’, highlighting an extensive range of opportunities to support health through planning.

The Sourcebook is also part of a suite of documents published by UN-Habitat on urban and territorial planning (UN-Habitat 2015, 2018a, 2018b) which is defined as ‘a decision-making process aimed at realizing economic, social, cultural and environmental goals through the development of spatial visions, strategies and plans and the application of a set of policy principles, tools, institutional and participatory mechanisms and regulatory procedures (UN-Habitat, 2015a, p. 2)’, that give rise to the spatial form, governance and management of human settlements.

UN-Habitat uses ‘urban and territorial planning’ as an attempt to alight on an internationally acceptable term covering recognisable policies, laws and mechanisms, and the practical actions stemming from these, that seek to exert spatial control over the occurrence, impact and distribution of public policy. In an international context, a number of different terms are in use in different countries, such as spatial planning, town and country planning or urban and regional planning. Each may have a distinct or defining characteristic stemming from national cultures and history (Dühr *et al*., 2010), however ‘urban and territorial planning’, as used by UN-Habitat refers to all of these (UN-Habitat, 2018b). In addressing health and its interaction with urban and territorial planning, the Sourcebook has had to take an even broader broach in its use of this term to include instances of weak, absent or disrupted planning systems, as one might find in informal settlements and marginal communities. It also extends the concept into the smaller scale interventions such urban and landscape design, and the more intimate scale of the street and public spaces.

The New Urban Agenda (United Nations, 2016) acts as a catalyst for urban and territorial planning as a delivery mechanism for value-laden public goods (Hague, 2018). For example, the effective implementation of the UN Sustainable Development Goals in cities and territories is explicitly acknowledged in paragraph 9. The New Urban Agenda also advocates a re-focusing of urban and territorial planning towards more people-centered practices. These two factors alone, though there are others, provide a strong foundation for centering ‘health’ as a key component for urban and territorial planning (Barton, 2009; Kleinert & Horton, 2016).

The International Guidelines on Urban and Territorial Planning (IG-UTP) were approved by UN-Habitat Governing Council through Resolution 25/6 in 2015 and are part of an enabling framework for addressing the challenges of urbanization. The intention is to assist countries and cities in managing urban demographic change and improving quality of life in new and existing urban settlements. The IG-UTP support different entry points and pathways for improving planning systems through a multi-level, multi-stakeholder, multi-sector perspective (UN-Habitat, 2015).

### Spatial Planning and Population Health

As we dip our toes into the Anthropocene (Steffen *et al*., 2011) humanity faces multiple health related crises of its own making. These are discernible at many scales: from the planet, through biomes and ecosystems, to cities, towns, and neighbourhoods (Grant *et al*., 2017). The COVID-19 pandemic has brought about a renewed focus on urban ‘public health’ as a science-based endeavour to shield disparate populations and communities from harms, prompted in this case by an infectious disease (Corburn *et al*., 2020; Scott, 2020).

Today the world’s population approaches 7.5bn, with at least 55% of people living in cities. Projections indicate that over the next decade the total urban population will rise from its current 4bn to over 6bn, with 66%, living in cities (UN, 2015). The ‘urban’ is the predominant habitat for humanity. We know that many diseases, both communicable and non-communicable, are associated with urban places (Prüss-Ustün *et al*., 2017)). Planning has, in general responded well to the health threats of communicable diseases and certain environmental issues, however there is a body of evidence and critical discourse indicating that current land-use and transport policies and practices in cities around the world can have direct negative health impacts, such as through road traffic injuries, air pollution, and support for physically inactive behaviours (Sallis 2016); Barton, 2009; Giles-Corti *et al*., 2016). These policies also influence other important determinants of health (such as access to jobs and education; community severance; climate change; and environmental degradation, to name a few) (Barton & Grant, 2006).

The health detracting relationships are well-documented – as are the many health supporting interventions that have potential to mitigate challenges and risks to health (Carmichael *et al*., 2012; Giles-Corti *et al*., 2016; Pinto *et al*., 2017). Indeed, historically planning has made enormous contributions to public health. Some point to the spatial epidemiological intervention of John Snow, whose ground-breaking testimony to policy-makers led to the handle of the Broad Street water pump to be removed in 1854 in response to a cholera outbreak in London (Snow, 1855), as the conceptual origins of that collaboration. As public health understanding of disease moved from miasma theory to germ theory, spatial interventions aimed at breaking the chain of transmission placed a spotlight on the efficacy of urban planning for health (Freestone & Wheeler, 2015). In England, US and Canada this can be seen in a progression of planning acts through the 19th and early to mid-20th Century that had strong public health goals such as sanitary and housing acts, slum clearance, and town/land planning. During this period the focus was on arresting environmental hazards, arresting causes of ill-health. In what Freestone and Wheeler refer to as the rebirth of the planning and health connection, starting in the 21st Century, the focus now also includes supporting environmental benefits to health such as opportunities for everyday activity (walking and cycling), contact with nearby nature and more compact development to support local amenities. This additional focus mirrors the focus of public health on the rise of non-communicable diseases such as obesity, stroke and heart diseases and mental health issues, where spatial factors are implicated in the causal web that underpins often-detrimental linkages between planning and health (Russo, 2011; Grant & Davis, 2018).

We must take it as a given that a healthy population is a fundamental goal of spatial development. Although due to epistemological fissures the evidence base is highly problematic (Rose, 1985; Grant & Davis, 2018), we can also surmise that costs of poor health (both treatment and loss to the economy) due to poorly functioning living environments might often outweigh the costs of planning and design more aligned with supporting better population health outcomes. If urban planning and design solutions are available, it would amount to collective negligence that we don’t place population health at the heart of urban and territorial planning. The literature and discourses surrounding health and urban spatial policy raise interesting questions, among them:

1. What leads us to build complex urban habitats that we know to be implicated in the prevalence of many of today’s most abundant non-communicable diseases (Rao *et al*., 2011; Sallis *et al*., 2016)?
2. Why do we continue to make spatial planning decisions that we know will compound health inequalities (WHO and UN Habitat, 2010; Rydin *et al*., 2012)?
3. How can we implement known solutions and avoid known mistakes to address communicable diseases and multiple morbidities in informal settlements (Oni *et al*., 2016; Ezeh *et al*., 2017)?

The answers are not simple, but these questions framed the thinking behind the Sourcebook. To a large degree, urban and regional planning systems have sought to maximize outcomes other than health, sometimes private profit from land, and usually economic growth, argued as a proxy for human welfare. The influence of major financial stakeholders in the development process itself, seeking to preserve absolute or relative advantage, should also not be underestimated. The ability of planning systems to even control development is also compromised in many places, as seen with informal settlements. This lack of urban and regional planning capacity at a global level, made more acute as urban populations expand, also plays a role (CAP, 2018 & 2020).

### Addressing Human Health at a Systemic Level

Planetary health has been defined as the health of human civilisation and the state of the natural systems on which it depends (Whitmee *et al*., 2015). Even where planning systems work well on their own terms, there will often be opportunities to better protect the environment through urban design and spatial planning. The places we create shape lifestyle choices of inhabitants that can force behaviours that exacerbate resource and energy use, these already place a burden on the planet’s ability to support healthy human life (MEA, 2005; Steffen *et al*., 2015; Romanelli *et al*., 2015). Thus, planetary health impacts too, are within the remit of ‘healthy urban planning’ (Barton & Tsourou, 2000).

Addressing this very broad human health agenda requires an acknowledgment to engage with complexity (Gatzweiler *et al*., 2018); the spatial design, physical form, and geo-locational parameters associated with human settlements, especially our towns and cities, emerge through the simultaneous intersecting actions of decision makers at many scales. The drivers that give rise to the physical and social determinants of health (and health equity) and the forms of urban governance that might exacerbate or mitigate those determinants are complex (Siri, 2016). Many are ‘upstream’, buried in the risk and investment decision approaches of developers (Black *et al*., 2018). As such, there can be no single response. We will need multiple interventions in urban development to address as many problematic elements as possible (Bai *et al*. 2016; Newell & Siri, 2016). Urban and territorial planning engages with complexity as a matter of course and, given a health ‘lens’, it is well-placed to generate effective solutions.

Public health has a tradition of evolution and change that has encompassed its nature, focus, and execution (Tulchinsky & Varavikova, 2014). With the eternal human ambition of reshaping conditions for better health at its heart, it has been acknowledging complexity and evolving systemic approaches (Lang & Rayner, 2012) since its earlier days. At this juncture, health as a sector, and public health as a speciality, must become more attentive to spatial determinants of health, better integrating them within policy and practice.

This may prove problematic as, even with planning and public health sharing a common ancestry, there has been a strong tendency for public health practice to become divorced from the systemics of spatial geographies and physical localities that are used as an evidence base for urban and territorial planning decisions (Sallis *et al*., 2006; Corburn, 2015). Indeed as far as evidence-based approaches are concerned, the epistemologies of public health and planning can even be seen as incompatible (Grant & Davis, 2018). Arguably, a diminished grounding in ‘place’ has allowed public health policy to drift toward a service delivery model, addressing individuals rather than populations affected by common spatial determinants. This can be faulted in economic terms as not the most effective way to treat those poor health outcomes that arise from environmental determinants. There may also be an effect of masking problems of health equity, when overall improvements in health are seen but together with a widening of the gap in health outcomes between urban sub-populations (CSDH, 2008). Environmental determinants leading to ill-health, such as poor air quality or obesogenic environments are concentrated in low income estates, slums and informal settlements. Rosy looking health statistics at national or city level can hide more granular health inequities (Rydin *et al*., 2012). In fact, health inequalities in cities were identified as a key factor undermining the achievement of the Millenium Development Goals (WHO and UN Habitat, 2010), with one-third of the world’s urban population living in slums and suffering much worse health, living and social conditions than other urban dwellers. This has led to a call to disaggregate health and health determinant data within the city to better target efforts.

As national economies plan a route back to economic stability following the COVID-19 pandemic, the immense role of public health and its connection to urban form has become apparent once again. This was reflected in Dr. Tedros Ghebreyesus’s intervention at the World Health Assembly in May 2020. In asking ‘ … what would the economic recovery look like if health mattered?’, he released the following statement:

The “lockdown” measures that have been necessary to control the spread of COVID-19 have slowed economic activity, and disrupted lives - but have also given some glimpses of a possible brighter future. In some places, pollution levels have dropped to such an extent that people have breathed clean air, or have seen blue skies and clear waters, or have been able to walk and cycle safely with their children - for the first times in their lives. The use of digital technology has accelerated new ways of working and connecting with each other, from reducing time spent commuting, to more flexible ways of studying, to carrying out medical consultations remotely, to spending more time with our families. Opinion polls from around the world show that people want to protect the environment and preserve the positives that have emerged from the crisis, as we recover. (WHO, 2020a, 1)

Outside the mainstream policy discourse, this sentiment was echoed by the Swedish climate activist Greta Thunberg a month later in the context of planetary health, expressing her hope that the crisis would open discussion about the urgency of action to limit health impacts related to climate change and environmental degradation now and in future (Rowlatt, 2020).

Recognising the immense challenges ahead, the public health and planning professions should not resign themselves to accepting that they cannot make a significant contribution to health in urban settlements. Indeed, they must rise to the challenge through the explicit incorporation of health within planning processes and practices.

### A Policy Led Settlement Planning Approach to Urban Health

Based on the preceding discussion, we think the time is right to strengthen policy collaboration between public health and urban and territorial planning. On the public health side making a distinct statement about the limits of health services in promoting health, the WHO used the Alma Ata Declaration to emphasise the holistic nature of individual health, stating ‘the action of many other social and economic sectors in addition to the health sector’ was required (WHO, 1978, Article 1). In 1986 it went further; ‘health promotion is not just the responsibility of the health sector’ in the Ottawa Charter for Health Promotion. Anticipating what has now been thoroughly fleshed out by an ever-expanding evidence base, the charter stated; ‘Our societies are complex and interrelated. Health cannot be separated from other goals. The inextricable links between people and their environment constitute the basis for a socio-ecological approach to health’ (WHO, 1986, p2). The thread of the WHO engaging with this aspect of urban health can be brought up to date with the Global Strategy on Health, Environment and Climate Change, recently adopted by WHO Member States (WHO, 2020b). This places urban and territorial planning at the core of the actions needed to fully integrate health into the decision-making process of all sectors and maximize societal welfare. The Strategy provides a vision and way forward for how the health community must respond to environmental health risks and challenges through 2030.

However, recognition that in population health still depends largely on people’s built environments, on influences outside the health sector having multiple direct and indirect influence on health outcomes (Barton & Grant, 2006) is not widespread, and we argue that action based on this recognition is even patchier. To support population health non-health sectors must be engaged in health governance, policy, and interventions (de Leeuw, 2017). The UN Sustainable Development Goals also make the case for health action beyond the health sector. Taken as an indivisible assemblage, many of the 17 goals contribute to the health outcomes of UN SDG 3 (Health and Wellbeing), especially UN SDG 11 (Sustainable Cities and Towns) in terms of urban health.

Practical experience of such policy collaborations already exists for example in Health in All Policies and Healthy Cities networks and programmes (WHO, 1990, 2013a). Such collaborations have led to many pockets of good practice; however, initiatives come and go according to the idiosyncrasies of funding, project champions, and political expediency (e.g. Hofstad, 2016). Large-scale, systemic change remains elusive. Indeed, an analysis of integrated health governance notes that effective arguments for cross-sectoral engagement are not enough to lead to change on their own. Systemic change requires the addition of clear conceptual heuristics or, more simply, clarity over how to best move forward (de Leeuw, 2017). The Sourcebook has been deliberately designed to contribute a Health in All Policies approach, providing readers with the conceptual scaffolding (de Leeuw & Peters, 2015) required to integrate health into many aspects of urban and territorial planning policy.

## Methodology and Project Development

As already stated, one purpose of this paper is to make the case for centering health as an input and outcome for good planning of urban environments, that was the focus of the preceding sections. In this section we turn our attention to an account how the Sourcebook was developed, and what shaped its final form.

The nature of the final product(s), at this stage a Sourcebook and a preliminary database, were not predefined but were determined during the project by the team as part of an iterative and reflexive process (Cunliffe, 2003) in response to a number of systematic evidence searches.

With the growing need for a more planning-based approach to urban health, a key question for the team was how best to (re)unite the public health and planning communities. Despite the publication of many resources and dissemination of tools and guidance for different situations, change has been slow at the national policy level or in customary practice. Therefore, a concern was how another publication from the long-standing collaboration between the WHO and UN-Habitat might support meaningful change. It was also clear that there was a need for better signposting, assessment and access to existing resources, and future ones as these became available.

### Initial Framing

Expert knowledge, confirmed later by searches, established that there were many published resources detailing ‘what to do’, with planning and design recommendations, but a deficit of material outlining ‘how’ to act. Early review of the current literature enabled the team to establish a framework of content for a final publication:

- Why action is needed

Articulating the urgency of the situation in terms of urban health, health equity and planetary health.

Listing the benefits for both urban and territorial planning and health, both benefit from a combined action.

- Who the key players are and the roles they perform

Providing clarity at different spatial levels of how to bring public health practice, skills and approaches into planning and design processes.

- How to act

Offering examples of effective actions to take in many different circumstances.

- When to act

Developing the concept of ‘entry points’ showing the multiple opportunities to bring a health approach into an urban and territorial planning proposition.

### A Different Kind of Guidance

In the early stages of the project, the intent was to prepare a straightforward guidance document (RMPU, 2017a & 2017b). However, during inception and literature review, the form of the final product was shaped by two arguments which shifted the approach and structure of the intervention.

Firstly, there was a wealth of context specific resources including guidance, but there remained a need for a how-to guide that was comprehensive in scope. The first comprehensive guide was published some 20 year ago, *Healthy Urban Planning: A WHO Guide to Planning for People* (Barton & Tsourou, 2000) covering nine policy areas; building regulations, housing policy, economic development, social support, open space, transport, energy, water and drainage and urban regeneration. Most subsequently published guidance has been more restricted in scope, either in terms of policy areas covered, geographic location or relevant scale. Such specificity of focus has led to the proliferation of guidelines, practical tools, checklists, evaluative and assessment systems, and other resources. Many of these are useful and practical in their own right, but often fail to address the broader change required in a systemic manner. This was confirmed through the literature searches.

Secondly, despite a widely acknowledged and growing policy agenda since 2000, there was not much evidence of mainstream action on the ground. Strategic urban and territorial planning carry on, for the most part, without any major attempt to systematically include health and health equity outcomes. It is critically acknowledged that several barriers impede mainstream translation of urban health research into policy and thence into action^1^ – productions of an additional piece of generic international guidance was perceived as unlikely to cut the Gordian knot and lead to significant progress. The authors have been aware of these barriers for some time and are either directly involved in the implementation, or funding, of several innovative initiatives that attempt to bridge this divide. The approach is to use transdisciplinary teams to link evidence, policy and action relevant to the spatial and built environment determinants of health (see box 1) – innovative in the field of urban health.

**Table:** 

**BOX 1:** A list providing six examples of recent and current transdisciplinary initiatives that address evidence, policy and action for urban health.
Moving Health Upstream in Urban Development Decision Making (UPSTREAM) (2016–19). United Kingdom. This research aimed to estimate the monetary impacts of built environment on human health and to explore barriers and opportunities for including health considerations in urban development with key decision-makers. (https://urban-health-upstream.info/). Salud Urbana en América Latina (SALURBAL) (2017–22). Latin America. The aims are understand how urban environments and urban policies impact the health of city residents throughout Latin America, to inform policies and interventions to create healthier, more equitable, and more sustainable cities. (https://drexel.edu/lac/salurbal/overview/). Complex Urban Systems for Sustainability and Health (CUSSH) (2018–22). China, Kenya, France & United Kingdom. Research to improve capacity for transformational changes in cities to meet environmental imperatives and improve the health and wellbeing by harnessing the benefits of sustainable policies and minimizing potential adverse consequences of global technological, environmental and social change. (https://www.ucl.ac.uk/drupal/site_complex-urban-systems/about-us). Pathways to Equitable, Healthy Cities (PATHWAYS) (2018–2022). China, Bangladesh, Ghana, United Kingdom & Canada. This aims to improve population health, enhance health equity and ensure environmental sustainability in cities through co-production of rigorous evidence with policy and civil society partners in cities. (http://equitablehealthycities.org/about-us/). Tackling the Root causes Upstream of Unhealthy Urban Development (TRUUD) (2019–2023). United Kingdom. Research to understand how prevention of non-communicable diseases might be fully considered and factored in to the decision-making of those in control of the quality of urban environments, focusing on major new infrastructure and critical, unresolved issues of governance (e.g. transport and air pollution, nature in cities). (https://www.bristol.ac.uk/population-health-sciences/projects/truud/project-summary/). Urban Health Initiative (UHI) (2019-onging). International. A programme that is developing a model and step-wise process for urban leaders to create demand for action for healthier cities by making the best use of local data, knowledge, competencies, and processes to include health in the development equation. The model process comprises six levels of urban transformation. These are mapping the current situation, adapting and applying health and economic tools, developing and testing scenarios, building engagement capacity, communication and outreach, and monitoring results and refining policy. The UHI process can be applied to address relevant entry points for healthier environments in the local context. (https://www.who.int/activities/implementing-the-urban-health-initiative).

These two arguments led to the decision to create two products – a document (Sourcebook) that would act as a framing and roadmap for action and a database of resources that can be updated as new resources are published. The Sourcebook is available online at www.who.int/publications/i/item/978924000317 and the database is in development, a preliminary example can be found in Appendix 2.

### Search Methodology

Literature searches were carried out in iterative tranches to find resources and shape the research programme. A formal public health systematic review (Jackson & Waters, 2005) was considered inappropriate for the task, given the focus on grey literature and known lack of assessments of efficacy. Details of search criteria can be found in Appendix 1. Searches covered English-language publications from 2008–2018. Other material was later accumulated by snowballing and through peer review recommendations but was assessed carefully for relevance.

The first tranche comprised seven Boolean keyword searches of standard databases, such as Web of Science, PubMed and the Journal of Urban Health. Advised by the results 14 searches further were carried out in institutional and thematic publications databases, such as the OECD, African Center for Cities, WHO and UN-Habitat repositories. At this point 9853 items had been found. Preliminary review of all titles or abstracts narrowed this to a database of 534 potentially relevant items. Advised by this remaining content, a second exclusion sweep eliminated articles written solely for academia and material that was only of historic interest. For final usability, resources that were not free to access or easily available were also excluded.

Outlines and document summaries were read to determine relevance, with retained documents categorised by date of publication, authority of source, audience, and theme. Searches of institutional databases were found to have a higher percentage of relevant documents. A specific exploratory search was also undertaken to find if there was any literature of ‘evaluations’ of urban health planning guidelines – no papers were found describing validated or proposed assessment tools for such guidelines.

Finally, records were added by snowballing from other known sources, recommendations from the research team and from peer reviewers of the first draft of the Sourcebook. These methods proved highly effective in identifying potentially relevant material. A final 622 items were reduced to 149 records following scans for duplicates (n = 30), and using the assessment criteria of relevance and quality. The full list of selection criteria can be found in Appendix 1 and full details of each of the 149 resources are provided (supplementary materials - table of resources). Inspection of the data over the initial 18 months of the project suggested that potentially useful new resources are published at a rate of roughly two every month. The implications of this are discussed later in the section on next steps.

### Final Selection and Representation of Resources in the Sourcebook

As discussed, the Sourcebook’s functionality is predicated on a theory of change that involves bringing awareness of ‘how to do’ health in territorial planning and to engage new audiences in new contexts. The resources are included in the Sourcebook as a means of providing examples of the wider determinants of health (such as including air quality, transport, housing, water, food and community) and to highlight multiple entry points for action. The final selection of resources to be included was carried out by the project team and tested through peer review of the final draft. Factors considered were:

1. Resources in some situations are plentiful, therefore the Sourcebook does not include every relevant, accessible, good quality resource for each particular need. Lack of inclusion for any specific resource does not imply any judgment on its quality.
2. Some resources are relevant across many different country situations and across multiple entry points. In order to include a maximum number of resources, resources tend to be referred to only once in the guidebook. This does not imply that they are not relevant in other situations. Where an entry point had many potential resources, some of those identified may appear in the database (supplementary materials - table of resources) and not the Sourcebook.

The authors recognise these as limitations in the printed version of the Sourcebook, for actors and decisions-makers looking for the most relevant resource in their own situation, the design of an online resource database should be able to overcome these limitations.

Of the final 149 selected items, only 69 were included in the Sourcebook. This was to ensure that the resources in the Sourcebook evenly represented coverage: for different stakeholders (as defined in the IG-UTP with the addition of public health); across different issues; for use in a wide spectrum of settlement types; and for cities and countries in a variety of economic and development contexts, using the World Bank income classification of countries.

#### Categories Used for Resources in the Sourcebook

Using a pragmatic approach to aid usability, each of the 69 resources has been allocated to one of 11 categories to indicate its application (Table 1). These categories emerged from an analysis of the nature of the resources themselves and the need to find a meaningful way to organise them.

**Table 1.** Categories of resource developed for the Sourcebook. Approaches and framework testing

ANALYTICAL TOOL	Tool for use in quantitative analysis
BRIEFING	Briefing for a specific approach
DESIGN GUIDE	Design process with rationale and instructions
EVIDENCE	Comprehensive subject specific evidence base
INITIATIVE	Reports on successful initiatives
NETWORK	Networks of policy and action
OVERVIEW	Broad overview of a subject area
SELF AUDIT	Tool to assist with baseline appraisal and analysis
TOOLKIT	Comprehensive stepwise instructions, with associated policy and evidence
TRAINING	Training packages
WEB RESOURCE	Source of online information and links for policy and action

#### Approches and framework testing

Several workshops with scenario based collaborative tasks were conducted to test this approach prior to the Sourcebook’s release in May 2020. They examined how the emerging approach could support knowledge exchange and capacity building activities in different contexts and with different audiences. Further details of the five workshops can be found in Appendix 2.

1. **Workshop 1**: Focus: Multisectoral approaches to urban health and wellbeing: The planning system assessment, Douala.
2. **Workshop 2**: Focus: Multi-sectoral initiatives for health in African cities: entry points approach to intersectoral action for NCD prevention, Cape Town.
3. **Workshop 3 & 4**: Focus: Interim findings and testing at international conferences with researchers and policy-makers, Kampala and Xiamen.
4. **Workshop 5**: Focus: Testing interim findings in an education and research setting, Taipei.

These workshops were used to test and refine the usability and application of concepts such as the wider determinants of health, the different levels of actors and decision makers, the spatial scales, and entry points. They were attended by a broad range of participants in terms of experience, profession, sector and discipline. The authors found those with a built environment background and those with a public health background were easily able to assimilate the concepts. They helped us refine details of how to best present them within the Sourcebook. In particular, the authors found the Health Map (Barton & Grant, 2006) a useful device to help the participants to find a common language during workshops tasks.

## What Have We Learnt?

The recent advancements in the knowledge and theory of planning for health are far from fully reflected in on-the-ground practice in urban environments. The sourcebook represents one step further along a pathway that includes development of the evidence base, high-level policy support and the publication of much specific guidance. Our research saw a need to focus on articulating a framework for action, showing how and when resources that are widely available can be put to work.

We choose to challenge current thinking by highlighting the following question:

“If the purpose of planning is not for human and planetary health, then what is it for?” (UN-Habitat and WHO, 2020 pXIII & p63)

The question is a provocation. It attempts to claim, or as some would say reclaim (Freestone & Wheeler, 2015), planning as a means for improving health and health equity. It sees public health practice as integral to urban and territorial planning- needing to be brought (back) ‘inside the tent’. We assert that public health must be an input for urban and territorial planning; if health is to be an outcome.

Especially for those in public health less conversant with planning processes, and their often complex and time delimited procedures, we developed a concept of ‘entry points’. The Sourcebook presents these in four clusters, with examples of each. Entry points:

1. by setting (e.g. public space, schools, streets, neighbourhoods);
2. by outcome (e.g. increase physical activity, increase to access nature, decrease health inequity);
3. by principle (e.g. road danger reduction, life-course strategies, health in all policies);
4. by sector (e.g. housing, local economy, food).

Our approach is deliberately aligned and compatible with prevailing global policy agendas, such as the New Urban Agenda, the United Nations Sustainable Development Goals, and the WHO’s aspiration for Universal Health Coverage. It offers a range of conceptual diagrams and visual aids to assist with communication, which can be easily adapted for local use. Collectively, these promote the use of a health ‘lens’ in planning and demonstrate the utility of that approach for a wide variety of stakeholder interests. Care was taken to transcend the traditional remit and limit implied by the term ‘urban and territorial planning’, so that we could address how health may be advanced through spatial planning and design - even where the planning system is weak or compromised, such as in informal settlements.

### Next Steps for Research

Comparing research into planning with that into urban health, we have two distinct disciplinary traditions. Given their disparate origins, these traditions hold substantially different epistemological interpretations, often-incompatible stances on the value of different forms of evidence (Rutter & Glonti, 2016; Rutter *et al*., 2017), and even conflicting ontologies (Grant & Davis, 2018). Moreover, some commentators are indicating that the next step is to better include political economy as a discourse (Greer *et al*., 2017) to urgently identify the lacunae in our policy systems that have allowed urban development to diverge so far from the best interests of human and planetary health.

Through a lens of systems theory, it is apparent that silos in scholarly thought have contributed to the planning/health divide and constitute an ongoing impediment to progress (Bai *et al*. 2016; Pongsiri *et al*., 2017; Gatzweiler *et al*., 2017). Therefore, a major priority must be the continued development of transdisciplinary research frameworks and practice domain (Lawrence & Gatzweiler, 2017). Its emergence is already evident in funding streams and initiatives (see Box 1) and the collaboration for this study. Programmes like the Wellcome Trust’s Our Planet, Our Health priority area and UK Research and Innovation’s Global Challenges Research Fund have promoted transdisciplinary research in recent years. The OECD recently released a major report (OECD, 2020) outlining concrete actions for governments, funders, universities and international organizations to support transdisciplinary research, evidence that this is attracting attention at the highest levels.

Given existing gaps in research at the interface of health and urban planning, a comprehensive research priority-setting exercise aimed at identifying understudied areas is key. Responding to this need, WHO has green-lighted the development of global research priorities for urban health as a prioritized global ‘public health good’ and recognized its importance to the delivery of WHO’s 13th General Programme of Work (WHO, 2019).

### Next Steps for Policy, Capacity and Practice

Although the Sourcebook is not primarily focused on policy, the authors hope that its publication and approach will spark further exploration of the critical role urban policy must play in planning for health. Among other things, policy can address deficits in institutional capacity and identify additional skills and competencies required for a more health-oriented approach. A key policy question may be how to create a demand and hence a market for healthy urban and territorial planning.

There are also significant challenges in technical training for both urban planning and built environment fields and forpublic health. Currenly these underplay the systemic nature of both problems and solutions in this shared arena, and thus fail to prepare new professionals to fully address the ramifications of the built environment on health.

### Where Next for Urban and Territorial Planning for Health?

The Sourcebook is just one more step on a long journey. It provides a useful roadmap to a labyrinth of resources. Such resources are relatively abundant and constantly growing. The next steps for our collaboration, and others who may want to join, will be to develop trainings and workshops showing participants how to find effective entry points. One challenge is that in many countries deficits in planning and health collaboration are further compounded by both a lack of institutional capacity and weak planning systems, especially in countries which are rapidly urbanising and are among the most vulnerable (CAP, 2018 & 2020).

Finally, in emphasising separate agendas for research, policy and practice, the preceding sections could give a false impression. The urgency and importance of this agenda calls for transformative action (Grin *et al*., 2010; Fudge *et al*., 2020), requiring ‘bridging communication’ between these three spheres of activity and the development of collaborative ‘learning capability’ (Fudge *et al*., 2020). To this end, we see it as vital that the ‘non-health’ professions develop ‘health literacy’.

A health-literate city is committed to intersectoral work across government because decision-makers in many sectors understand the high relevance of health and seek health co-benefits and synergy in their policies in cooperation with the health sector. (WHO, 2013c , p. 29)

Our collaboration will seek to invest in communities of practice (Bai *et al*., 2019). Next steps for ourselves and others who may want to join us include:

1. **Joint initiatives for capacity building.** Including the creation of training packages, knowledge platforms, applied research, tools for application and influencing for policy change.
2. **Identification of knowledge and policy gaps.** Including gaps in the current curriculum for built environment and public health practitioners (basic education, advanced training, certification programs) and in the current policy environment.
3. **Mutual technical support in research/policy/practice.**Including gathering knowledge and data, developing advocacy, assisting in the development of tools and guidelines for their use.

Global and regional communities of practice are critical, recognising that urban health and healthy cities are high on the agenda but insufficiently supported by capacity on the ground. Locally produced resources, but with potential for more widespread application, are being published all the time. A community of practice could develop a database, (supplementary materials - table of resources), and provide up-to-date access to new resources, broadening their reach through setting them within the framework of entry points. It could also support the development of training and workshops, assisting with assessments of planning contexts (including regulatory frameworks, programs and processes) through a health lens.

The next steps for urban and territorial planning for health must be anchored in a shared vision for a healthier future and the urgency of action, considering the risks to urban health uncovered by the COVID-19 crisis (Ellis et al., 2021). WHO’s ‘Manifesto for a healthy and green COVID-19 recovery’ (WHO, 2020a) places urban and territorial planning at the core of the action needed to revert the ‘escalating damage to the ecological systems that sustain all human health and livelihoods’, including prescriptions directly related to urban and territorial planning. It names building healthy, liveable cities; protecting and preserving the source of human health and nature; and promoting healthy, sustainable food systems.

The evidence base is robust, and resources are available for a wealth of scales and situations. Urban and territorial planning must be rapidly developed as a core activity in public health and *visa versa*. The Sourcebook, and actions stemming from its ‘how-to’ approach, could play an important role in unlocking the next stage in urban and territorial planning for public health.

## Supplementary Material

Supplementary Materials - Table of Resources

## Note

1. In the route from new knowledge to policy to action there are three main hurdles where attention needs to be focused.Firstly, it is a common fallacy to assume that once science reveals new knowledge policymakers take notice. The World Health Organization has referred to this as the science-policy gap (WHO, 2008).Secondly, the existence of a policy-action gap has also long been recognized. Experience and knowledge, which may be a driver for new policy, has been found to rarely translate smoothly into improved action at organizational or systemic levels, as highlighted in implementation studies from the 1960s onward (Dror, 1968; Pressman & Wildavsky, 1975; Dunsire, 1978). Policy-making institutions often mistakenly assume that the establishment of committed policy then yields the desired action; whereas, in practice, outcomes may appear, but even from the policy makers’ perspective they are a long way short of the original intentions (Barrett & Fudge, 1981).Thirdly, transdisciplinary complexity (Lawrence & Després, 2004), such as that underlying applying health outcomes to urban and territorial planning, further complicates the pathway from knowledge to policy to action. Urban and territorial planning must be evidence-informed, with an evidence base that embraces complex, multiple, upstream, population-level actions and outcomes. It is far easier for public health policy makers to prioritise and focus on more-easily-assessed individual-level interventions, despite the widening recognition of the need for system-level responses (Rutter *et al*., 2017).
