# Supplementary material for: Supporting a Healthy Planet, Healthy People and Health Equity through Urban and Territorial Planning
Source: Plan Pract Res. 2022 Jan 23;37(1):111–30. doi: 10.1080/02697459.2021.2000144 (PMC8827622; doi:10.1080/02697459.2021.2000144)
Supplement: Supplementary Materials - Table of Resources [file CPPR_A_2000144_SM8278.pdf]

Supporting a healthy planet, healthy people and health equity through urban and territorial planning

Supplementary Materials – Table of resources

The supplemental material contains a table of 149 selected resources.
This comprises the 69 resources which appear in the published Sourcebook (UN-Habitat & WHO 2020) and an additional 79 that were selected as relevant but not included (see paper for discussion on inclusion criteria).

For further information on the searches undertaken and how resources were selected or the published version see Appendix 1.

Reference:
UN-Habitat and WHO, 2020. *Integrating health in urban and territorial planning: A sourcebook*. Nairobi: UN-Habitat and Geneva: World Health Organization

The nature of the fields for each record in the table are shown in the table below.

Fields

Record built from citation database, snowball or peer recommendation.	Text	Item record as found in citation database. Contains with title and url link. May also contain Author and date depending on database
Resource in Sourcebook?	Y or N	Included within the published Sourcebook
Sourcebook Ref. No.	No.	Resource reference number if in Sourcebook
Type of resource	Text	One of 11 categories allocated to the resource (see table 5, Annex 1)
Key entry point(s)	Text	As outlined in the Sourcebook
Author or Publisher	Text	
Date	Date	Date of publication
Description	Text	Text as in Sourcebook where relevant or in draft for the non-included items
Audience	Text	Text as in Sourcebook (otherwise blank)
Good for these situations	Text	Text as in Sourcebook (otherwise blank)
Search Id	Text	Reference indicating how the resource was identified

Keys to fields using abbreviations

Categories of resource developed for the Sourcebook

**Type of resource** (as in referred to the Sourcebook)	**Description**
**Analytical tool**	Tool for use in quantitative analysis.
**Briefing**	Briefing for a specific approach.
**Design guide**	Design process with rationale and instructions.
**Evidence**	Comprehensive subject specific evidence base.
**Initiative**	Reports on successful initiatives.
**Network**	Networks of policy and action.
**Overview**	Broad overview of a subject area.
**Self audit**	Tool to assist with baseline appraisal and analysis.
**Toolkit**	Comprehensive stepwise instructions, with associated policy and evidence.
**Training**	Training packages.
**Web resource**	Source of online information and links for policy and action.

Identification of resources

**Total from each search. (details of searches are in appendix 1)**	**Number of resource records in spreadsheet**
ACfC	1
HUDU	4
IIED	6
OECD	1
peer	40
PubMed_S1	5
PubMed_S3	3
snowball	51
ULI	7
WHO_S1	19
WHO_Slim	7
WoS	4
**TOTAL**	**148**

Record built from citation database, snowball or peer recommendation.	In sourcebook?	Sourcebook Ref	Type of resource	Key entry point(s)	Author or Publisher	Date	Description	Audience (text as in sourcebook, otherwise blank)	Good for these situations (text as in sourcebook, otherwise blank)	Search Id
Public Health England, Spatial Planning for Health: An Evidence Resource for Planning and Designing Healthier Places, 2017. Available online at https://assets.publishing.service.gov.uk/government/uploads/system/uploads/attachment_data/file/625568/Spatial_planning_for_health_an_evidence_resource.pdf	Y	1	Evidence	community; policy; places;	Public Health England	2017	The primary target audience of this tool is local public health professionals, but also planners working in local authority settings. The review identifies, critically appraises and summarizes existing review-level evidence of associations between the built and natural environment and health outcomes. The review is centred on five aspects of the built and natural environment: neighbourhood design, housing, healthier food, natural and sustainable environment, and transport.	The findings are designed to be suitable for both public health practitioners and planning professionals, facilitating two-way between disciplines.	Arising from the English context, this resource would be valuable for many cities and towns in the world where people's choices for healthier lifestyles are limited by the built environment.	HUDU
Urban Land Institute. Building Healthy Places Toolkit: Strategies for Enhancing Health in the Built Environment. Washington, DC: Urban Land Institute, 2015 https://centerforactivedesign.org/buildinghealthyplacestoolkit	Y	2	Toolkit	health outcomes; urban design;	Washington, DC: Urban Land Institute	2015	Resource and reference document providing specific evidence-supported design and programming recommendations that relate to health.	For built environment professionals, developers and public health practitioners who are seeking to shape buildings and projects in ways that enhance and promote health.	Arising from the USA, this resource would be valuable for guiding development in cities and towns in the world where there is an ambition to optimize health outcomes.	ULI
UN-Habitat. Urban-Rural Linkages: Guiding Principles. Framework for Action to Advance Integrated Territorial Development. 2018. https://urbanrurallinkages.files.wordpress.com/2019/09/url-gp-1.pdf	Y	3	Overview	peri-urban; participation; rural;	UN-Habitat	2018	A multilevel, multi-stakeholder guidance framework and tool to strengthen urban-rural linkages in national and subnational policies and programmes. Protection and promotion of health by balancing urban, peri-urban and rural health challenges are included in the guiding principles to provide social protection and do no harm.	Policy-makers at all governance levels, programme managers, private sector and civil society actors and implementation partners of local and subnational governments.	Incorporating into public policy and programme provision for social services across the urban-rural continuum such as coordinated health, nutrition and sanitation plans, reduction of spatial and social inequities in quality health services, and mainstreaming efforts to create healthy and safe environments in integrated, resilient and sustainable UTP.	peer

Record built from citation database, snowball or peer recommendation.	In sourcebook?	Sourcebook Ref	Type of resource	Key entry point(s)	Author or Publisher	Date	Description	Audience (text as in sourcebook, otherwise blank)	Good for these situations (text as in sourcebook, otherwise blank)	Search Id
The role of cities in improving population health: International insights Chris Naylor David Buck 2018 The King's Fund: London https://www.kingsfund.org.uk/publications/cities-population-health	Y	4	Overview	governance; leadership; cities;	Naylor C, Buck D, London: The King's Fund	2018	Examines cities as playing a growing role in population health improvement with the enormous potential to be health-generating places. The report is based on 50 interviews with leaders from 14 cities and draws on international case studies. It concludes that improving population health depends on many factors, including: coordinated action at multiple levels, bold political leadership, empowered citizens, effective use of planning powers and regulatory measures.	Those wanting to explore how cities and their leaders can maximize opportunities to improve population health including public health practitioners.	A wide range of cities covering high-income countries and LMICs.	snowball
UN-Habitat. UN-Habitat planning law assessment framework. 2017. https://unhabitat.org/planning-lawassessment-framework	Y	5	Self audit	planning law; assessment; participation; legal; cities;	UN-Habitat	2017	The planning law assessment framework is a self-assessment tool to be used during focus groups for the preliminary identification of strengths and weakness of an urban planning system. The framework looks at all the laws, regulations and decrees applicable in a city, enacted at different levels. It takes into account only black letter law but will stimulate the discussion on eventual discrepancies. This process is useful to change mind-sets, learning process, to create constituency creation; it could be the first step to pursue a reform process.	Wide range of users of planning law in a given jurisdiction. It is recommended to have experts and specialists in planning law leading the assessment. The methodology should take the form of focus groups, interviews and expert group meetings.	Rapid assessment to identify the strengths and weaknesses of an urban planning law and guiding a process to agree on actions that are needed to address the identified gaps.	peer
African Centre for Cities, Cities Alliance, Urban LandMark and UN-Habitat. Reforming urban laws in Africa: a practical guide. 2017. https://www.africancentreforcities.net/wp-content/uploads/2017/06/ULR-Report_FINAL_LR.pdf	Y	6	Self audit	planning law; assessment; participation; legislation; cities;	African Centre for Cities, Cities Alliance, Urban LandMark and UN-Habitat	2017	This guide focuses on the law-making implementation challenge: how to make progress with an intention to make better laws for towns and cities in Africa. It proposes an approach to urban law-making that is grounded in an understanding of the local context. Produced largely by the African Centre for Cities, experience from senior practitioner researchers of urban law-making is combined to provide a practical guide for officials and other practitioners. The guide is also available in French and Portuguese.	Wide range of interest groups and officials.	Better understanding of the laws determining how cities work, how the legal system works and how the government works at different levels.	snowball

Record built from citation database, snowball or peer recommendation.	In sourcebook?	Sourcebook Ref	Type of resource	Key entry point(s)	Author or Publisher	Date	Description	Audience (text as in sourcebook, otherwise blank)	Good for these situations (text as in sourcebook, otherwise blank)	Search Id
UN-Habitat. Slum Upgrading Legal Assessment Tool. 2019. unhabitat-ig-utp@un.org	Y	7	Self audit	planning law; assessment; participation;legislation; cities; slums; informal settlements;	UN-Habitat	2019	This legal assessment tool provides urban managers and other stakeholders with a framework to understand how and if their legal and regulatory framework supports participatory citywide slum upgrading or not. It is a self-assessment tool to be used during focus groups, to identify strengths and weakness of the current urban planning system and guide opportunities for citywide slum upgrading. It can provide a clarifying process to make clear what frameworks might need to be revised as part of a longer term reform process and also serve as an entry point to change mind-sets and build capacity around legal and regulatory frameworks for participatory citywide slum upgrading.	Urban managers and other key stakeholders.	Robust domestic legal analysis, supported by a participatory discussion which outlines the strengths and opportunities or impediments of current legal and regulatory frameworks for slum upgrading.	peer
Designing and Implementing Citywide Slum Upgrading Programs: A Training Module Companion. UN-Habitat 2013 https://unhabitat.org/books/designing-and-implementing-street-led-citywide-slum-upgrading-programmes-a-training-module-companion/	Y	8	Training	slums; informal settlements; streets; participation; upgrading;	UN-Habitat	2013	This training module introduces a practical strategy for improvement of streets through citizen participation as a strategic spatial intervention for citywide slum upgrading. The approach fosters incremental improvement of the physical and socioeconomic conditions in slums and informal settlements and promotes urban regeneration, transformation and their integration into the overall city planning agenda.	Especially useful for participatory planning, advocacy and case making.	Wide range of actors and decision-makers, including public health practitioners.	snowball
Addressing health of the urban poor in South-East Asia Region: challenges and opportunities. World Health Organization, Regional Office for South-East Asia. 2011 http://www.who.int/iris/handle/10665/204753	Y	9	Overview	urban poor; land use; food; housing; transport; energy;	WHO Regional Office for South-East Asia	2011	An overview of the health of the urban poor in Member States of the WHO South-East Asia Region focusing on health protection. It addresses the built environment determinants of health including land use, food access, housing, transport and domestic energy sources.	Policy-makers, programme managers and public health and planning professionals.	Making the case for a strategic framework of multi-sectoral action and showing the connected urban action needed covering a range of SDG goals and targets, including climate change. Applicable to slums and the problems of rapid and unplanned urbanization.	WHO S1

Record built from citation database, snowball or peer recommendation.	In sourcebook?	Sourcebook Ref	Type of resource	Key entry point(s)	Author or Publisher	Date	Description	Audience (text as in sourcebook, otherwise blank)	Good for these situations (text as in sourcebook, otherwise blank)	Search Id
UN-Habitat. Rapid Planning Studio – concept note. Rapid Planning Studio – workshop schedule. 2016. unhabitat-ig-utp@un.org	Y	10	Toolkit	participation; legislation; urban design; finance;	UN-Habitat	2016	Rapid Planning Studio aims at strengthening planning capacity of participating municipalities for sustainable urban development and providing a clear planning methodology and an actionable roadmap to supply serviced land for rapid urban growth. A workshop integrating the three basic pillars – urban legislation, urban finance and economy, and urban planning and design – of sustainable urban development simulating a full planning process in a rapid, 3-day format.	Municipal staff and community activists.	Applicable worldwide. Harnessing the knowledge, talents and energies of all parties to discuss citywide urban analysis and profiling, strategic planning, urban transformation and public spaces, focusing specifically on answering the challenges of participating municipalities regarding planned city extensions.	peer
BENDING THE CURVE ON URBAN DIABETES: New research approaches and innovative interventions for tackling diabetes in your city. CitiesChangingDiabetes, 2017. http://www.citieschangingdiabetes.com/content/dam/cities-changing-diabetes/magazines/CCD-BriefingBook-2017-BendTheCurveOnUrbanDiabetes.pdf	Y	11	Initiative	health; diabetes;	Cities Changing Diabetes	2017	This briefing presents highlights from research and diabetes action pilots in eight cities worldwide. The approach demonstrates how urban planning needs to be embedded as a solution to this health challenge. The focus is obesity, the single most significant driver of diabetes. Pitched against an objective to hold the rise of diabetes prevalence to 10% globally; a model is presented on what must be done to reduce obesity by 25% globally by 2045.	Policy-makers, programme managers and public health and planning professionals.	Setting goals and establishing an action plan for response to a rise in diabetes and offering an approach for cities, towns and communities to set goals and take action.	snowball
STIPO, The Netherlands. City at eye level. https://thecityateyelevel.com/	Y	12	Initiative	public space; public realm; streets; local economy; shops;	STIPO, The Netherlands.	undated	This is a worldwide programme with many partners. The website has many resources including open source books, some put together in partnership with UN-Habitat.	Community, businesses, the public sector and public health practitioners.	Shared working through seeing a place through the eyes of a child.	snowball

Record built from citation database, snowball or peer recommendation.	In sourcebook?	Sourcebook Ref	Type of resource	Key entry point(s)	Author or Publisher	Date	Description	Audience (text as in sourcebook, otherwise blank)	Good for these situations (text as in sourcebook, otherwise blank)	Search Id
Population Health Institute, University of Wisconsin-Madison. Build public & political will. Activity 6 – Act on what's important: key activities; county health rankings. https://www.countyhealthrankings.org/key-activities/18392#key-activity-6	Y	13	Training	leadership; participation; communication; health literacy;	Population Health Institute, University of Wisconsin-Madison.	undated	An online course delivered as a suite of advice and frameworks for promoting policy, systems and environmental changes to improve community health for the long term. The section featured here specifically looks at how to develop public and political will.	Policy-makers, programme managers and public health and urban planning professionals.	Advocacy and developing public and political support especially for disadvantaged and marginalized communities.	snowball
Department of Design and Construction and others, 'Active Design Guidelines: Promoting Physical Activity and Health in Design', New York City, 2011. Available at https://centerforactivedesign.org/guidelines/	Y	14	Design guide	health literacy; city centres; phsical activity; obesity; walking; active travel;	New York City	2011	Citywide manual for modern city centres promoting physical activity and health.	Everyone involved with built environment design, including architects, planners, urban designers, transportation planners and landscape architects and building sponsors in public or private sectors.	Anywhere in the world, especially city centres with potential to promote physical activity and well-being through the design of the built environment.	ULI
Noncommunicable diseases: what municipal authorities, local governments and ministries responsible for urban planning need to know. 2016. World Health Organization and United Nations Development Programme. Available online at http://www.who.int/iris/handle/10665/250228.	Y	15	Briefing	urbanisation; NCDs; leadership; cities;	WHO & UNDP	2016	A short advocacy briefing focusing on addressing NCDs through urban planning.	Municipal authorities, local governments and ministries responsible for urban planning.	Dealing with a broad range of health challenges and widely applicable cities at any stage of development. Support for HiAP and SDG approaches.	WHO S1
Health in all policies: training manual World Health Organization 2015 http://apps.who.int/iris/bitstream/handle/10665/151788/9789241507981_eng.pdf	Y	16	Training	HiAP; participation; health literacy;	WHO	2016	A comprehensive training manual for HiAP work with the aim for workshop participants to gain the most from a learning-by-doing, participatory approach.	Training routeways are provided for politicians and senior policymakers (2 days) and for policy and programme managers (3 days).	Establishing a shared approach through the guided group discussions and activities which allow participants to build on and apply their knowledge and experience. Applicable worldwide.	peer

Record built from citation database, snowball or peer recommendation.	In sourcebook?	Sourcebook Ref	Type of resource	Key entry point(s)	Author or Publisher	Date	Description	Audience (text as in sourcebook, otherwise blank)	Good for these situations (text as in sourcebook, otherwise blank)	Search Id
Global Public Space Toolkit: From Global Principles to Local Policies and Practice UN-Habitat 2015 https://unhabitat.org/wp-content/uploads/2015/10/Global%20Public%20Space%20Toolkit.pdf	Y	17	Toolkit	public spaces	UN-Habitat	2015	The structure chosen for this work rests on three elements: why, what and how: the case for public space; goals, constraints, principles and policies; and turning good principles into actions. The toolkit has been designed to be available to all and easily accessible. The text is illustrated by brief quotes and practical examples of cases on past or on-going public space initiatives. A web-based version, which is intended to grow and be enriched with contributions from local and global actors, can be found at: www.urbangateway.org/publicspace	City authorities with interest for practitioners and community activists.	Widely applicable including informal settlements in LMICs.	snowball
UN-Habitat. Turning spaces into places – handbook. 2013. https://unhabitat-kosovo.org/un_habitat_documents/turning-spaces-into-placeshandbook/	Y	18	Design guide	public spaces	UN-Habitat	2013	The handbook outlines key underlying design characteristics of "good public places" by introducing some global concepts and local examples. It aims to stimulate discussion, generate ideas, collective thinking and raise awareness amongst decision-makers about placemaking versus space-maintaining. The handbook develops some design principles and techniques and contains inspiring examples of what can be achieved.	Mayors, urban planners, developers, and all those concerned with the development of towns and cities, and with the quality and importance of public spaces.	Explaining what placemaking is, and how placemaking impacts people's lives and how it can be achieved.	peer
Ciclovias Recreativas / Open Streets https://cicloviarecreativa.uniandes.edu.co/english/introduction.html Guidance from Universidad de los Andes on how to plan, implement and evaluate Ciclovias Recreativas / Open Streets	Y	19	Web resource	public spaces	Universidad de los Andes, Colombia	undated	Guidance and case studies from Ciclovía Recreativa. This is an initiative for the temporary opening of streets to residents to enjoy them as safe and pleasant spaces for walking, jogging, skating or cycling. Ciclovía Recreativa projects differ from permanent bike routes because they promotes temporary spaces where the principal use is not for transport, but for recreation. In general, Ciclovía Recreativa occurs on a fixed day of the week (often Sundays and in some cases on holidays) and has an average duration of 6 hours. Also available in Spanish.	Urban planners, communities and public health practitioners.	Planning, implementing and evaluating Ciclovía Recreativa/Open Streets initiatives.	peer

Record built from citation database, snowball or peer recommendation.	In sourcebook?	Sourcebook Ref	Type of resource	Key entry point(s)	Author or Publisher	Date	Description	Audience (text as in sourcebook, otherwise blank)	Good for these situations (text as in sourcebook, otherwise blank)	Search Id
A new strategy of sustainable neighbourhood planning: Five Principles UN-Habitat 2014 https://unhabitat.org/a-new-strategy-of-sustainable-neighbourhood-planning-five-principles-0	Y	20	Briefing	neighbourhoods	UN-Habitat	2014	In supporting sustainable neighbourhoods, these principles seek to: promote high-density urban growth, alleviate urban sprawl and maximize land efficiency; promote sustainable, diversified, socially equal and thriving communities in economically viable ways; encourage walkable neighbourhoods and reduce car dependency; optimize use of land and provide an interconnected network of streets which facilitate safe, efficient and pleasant walking, cycling and driving; foster local employment, local production and local consumption; provide a variety of plot sizes and housing types to cater for the diverse housing needs of communities, at densities which can ultimately support the provision of local services.	Local decision-makers, planners, public health professionals and communities.	Worldwide application for advocacy and communication of key health principles for local neighbourhood planning, design and regeneration.	snowball
BC Centre for Disease Control. Healthy Built Environment Linkages Toolkit: making the links between design, planning and health, Version 2.0. Vancouver, B.C. Provincial Health Services Authority, 2018. Toolkit Available at http://www.bccdc.ca/health-professionals/professional-resources/healthy-built-environment-linkages-toolkit	Y	21	Toolkit	neighbourhoods	Vancouver, BC: Provincial Health Services Authority	2018	This 80-page toolkit is very comprehensive and describes how population health is influenced by the design of our neighbourhoods, housing, transportation systems, natural environments and food systems. It brings together research-based key messages that correlate land-use planning decisions, impacts on the built environment and population health. Health professionals and others working to assist local governments and provide well-informed and credible recommendations will find this resource useful to draw from. Also available in French.	The toolkit has been written for health professionals to assist them in articulating well-informed and credible responses within local government planning processes and decision-making. It can also be used by other stakeholders, such as planners, who may find the health evidence provided is helpful to build the case for healthier placemaking.	The toolkit has been created to generate conversations and real-world adaptation by outlining a rationale for why the built environment is important for health. Although coming from a high-income country, the principles are widely relevant and can be adapted to many global situations.	snowball

Record built from citation database, snowball or peer recommendation.	In sourcebook?	Sourcebook Ref	Type of resource	Key entry point(s)	Author or Publisher	Date	Description	Audience (text as in sourcebook, otherwise blank)	Good for these situations (text as in sourcebook, otherwise blank)	Search Id
Corburn J and A Sverdlik (2016). Informal Settlement Upgrading and Health Equity, in J. Corburn and L. Riley, eds., Slum Health: From the Cell to the Street, Univ of California Press; 2016. https://www.researchgate.net/publication/308674541_Urban_Informal_Settlement_Upgrading_and_Health_Equity	Y	22	Briefing (book chapter)	neighbourhoods; informal settlements	Corburn & Sverdlik	2016	This text looks at upgrading initiatives of informal settlements in the Global South and the health implications. There is a discussion of how urban health inequalities can be reduced by responsive governance and participatory, multi-sectoral upgrading initiatives in informal settlements (or slums) and of pathways between upgrading and health equity and a critical review of a range of published evaluation. It concludes by proposing more nuanced, mixed methods evaluations that can better reveal how upgrading projects can influence health and support well-being in informal settlements.	Public health practitioners and regeneration interests.	Informal settlements.	WHO_Slum
Healthy High Streets: Good place-making in an urban setting. 2018. Public Health England. Available online from https://assets.publishing.service.gov.uk/government/uploads/system/uploads/attachment_data/file/699295/26.01.18_Healthy_High_Streets_Full_Report_Final_version_3.pdf	Y	23	Evidence	high street;s	Public Health England	2018	This report synthesises the latest and most relevant evidence. Specifically, the report examines how important features of the high street can positively impact on social cohesion, and on mental and physical health. It provides street design principles.	Local decision-makers, planners, urban designers, landscape architects, public health practitioners and other professionals involved in creating high streets.	Making high streets more inclusive, safe and healthy and that promote social integration, particularly in areas of high deprivation. Applicable to any highly built-up environments in cities or neighbourhoods; smaller high streets in suburbs are also covered.	snowball
UN-Habitat/Institute for Transportation and Development Policy. Streets for walking and cycling: designing for safety, accessibility, and comfort in African cities. 2018. https://www.itdp.org/publication/africa-streets-walking-cycling/	Y	24	Design guide	local streets	UN-Habitat/Institute for Transportation and Development Policy	2018	The guide emphasizes designing for safety, accessibility and comfort in African cities. These cities are fundamentally walking and cycling cities, but often lack the infrastructure and design to make these activities safe and comfortable. This design book provides detailed guidance on how to address these issues.	City planners, engineers and architects across Africa. The findings are designed to be suitable for both public health practitioners and transport and planning professionals, facilitating two-way communication between disciplines.	Better design of roads, provision of safe and more convenient pedestrian crossings and separation between high-speed vehicles and people to make walking and cycling safer.	peer

Record built from citation database, snowball or peer recommendation.	In sourcebook?	Sourcebook Ref	Type of resource	Key entry point(s)	Author or Publisher	Date	Description	Audience (text as in sourcebook, otherwise blank)	Good for these situations (text as in sourcebook, otherwise blank)	Search Id
UN-Habitat. Streets as tools for urban transformation in slums: A street-led approach to citywide slum upgrading; UN-Habitat 2014. https://unhabitat.org/books/streets-as-tools-for-urban-transformation-in-slums/	Y	25	Design guide	streets; slums; informal settlements;	UN-Habitat	2014	The focus is on the streets and urban layout of settlements, as the drivers of transformation and regeneration. It reviews citizens' involvement in participatory planning and re-emphasises the importance of mapping through participatory enumeration and locally acceptable forms of social and physical mapping. It covers: basic infrastructure provision, e.g. water supply, sanitation, drainage; land allocation for resettlement and new housing provision; and ensuring security of land tenure within slums, ultimately leading to regularization and legalization. The approach is an incremental one to integrating slums using plenty of examples.	City authorities, community activists and public health practitioners.	Informal settlements in LMICs.	WHO_Slum
Ciclovía Recreativa/Universidad de los Andes, Colombia. Ciclovia Recreativa in Latin America and Open Streets in South Africa. https://www.nationalgeographic.com/environment/2019/03/bogotacolombia-cicloviabans-cars-on-roadseach-sunday/ and https://openstreets.org.za/	Y	26	Network	local streets	Ciclovía Recreativa/Universidad de los Andes, Colombia	2014	What started as an initiative in Bogotá, Colombia, then spread to hundreds of towns and cities in many countries. The activity is a time-limited period (weekly and/or on major public holidays) when specific major roads are closed to traffic so that residents have the space for jogging, running, skating, cycling and aerobics. It started as a way of encouraging fitness but now its social benefits of providing spaces to meet with friends, family and fellow city dwellers of all ages are also recognized. The name Ciclovía arises from the seven cycle-friendly routes in Bogotá covering 121 km that the first initiative encompassed.	Mayors and local politicians, local people and public health practitioners.	Bringing activity and social connections into cities without major infrastructure costs.	peer
Urban green spaces: A brief for action, WHO EURO, 2017. https://www.euro.who.int/__data/assets/pdf_file/0010/342289/Urban-Green-Spaces_EN_WHO_web3.pdf	Y	27	Briefing	green spaces	WHO Regional Office for Europe	2017	This briefing presents the key findings of a review of research evidence and practical case studies on urban green space interventions and provides implications for practice. It covers urban green spaces and their benefits, and planning and design involving the community and stakeholders. It promotes monitoring and evaluation. It also describes potential risks and challenges to be considered and avoided with a set of key messages and further reading.	To support urban policy-makers and practitioners.	Global application for cities, towns and local neighbourhoods when designing urban green spaces to maximize social and health benefits.	peer

Record built from citation database, snowball or peer recommendation.	In sourcebook?	Sourcebook Ref	Type of resource	Key entry point(s)	Author or Publisher	Date	Description	Audience (text as in sourcebook, otherwise blank)	Good for these situations (text as in sourcebook, otherwise blank)	Search Id
UN-Habitat. Promoting non-motorized transport in Asian cities: policymakers' toolbox. 2013. https://unhabitat.org/sites/default/files/download-manager-files/Promoting%20NMT%20in%20Asian%20CitiesS.pdf	Y	28	Toolkit	physical activity	UN-Habitat	2013	This is a comprehensive briefing and design manual with several practical survey and audit tools. The health focus is improving air quality. However, a shift to cycling and walking will increase physical activity. Contains case studies and options for solutions.	Transport and public health professionals working in or with cities.	Auditing and action planning in cities in LMICs that are facing a rise in personal motorized transport with the consequent increase in health impacts from pollution.	peer
Global action plan on physical activity 2018–2030: more active people for a healthier world. Geneva: World Health Organization; 2018. http://www.who.int/ncds/prevention/physical-activity/global-action-plan-2018-2030/en/	Y	29	Initiative	physical activity	WHO	2018	A widely applicable plan for action to support everyday physical activity developed through a worldwide consultation process involving governments and key stakeholders across multiple sectors including health, sports, transport, urban design, civil society, academia and the private sector.	Action points for city leaders, stakeholders and Member States.	Focusing action on what will best support active lives. Presents the urban environment as an essential part of active lives. Provides five actions for creating active environments broken down into steps for each stakeholder group.	peer
Clean Household Energy Solutions Toolkit (CHEST) WHO Online http://www.who.int/airpollution/household/chest/en/	Y	30	Toolkit	air quality; energy; housing	WHO	undated	For clean and safe interventions in the home. Helps health sector professionals and policy-makers implement the recommendations found in WHO guidelines on indoor air quality and household fuel combustion. It provides resources to guide the energy planning process, using evidence from WHO databases and training materials.	Public health professional and planners working in countries with health risks from household fuel combustion.	Contains tools for assessment of the current state of household energy use, air pollution and health impacts. It facilitates the design of policies that promote the adoption of clean household energy at a local, programmatic or national level.	peer
WHO, Climate and Clean Air Coalition, UNEP, World Bank. BreatheLife global campaign. https://breathelife2030.org/breathelife-cities/	Y	31	Initiative	air quality; energy; housing; tranport;	WHO, Climate and Clean Air Coalition, UNEP, World Bank	undated	A network programme for cities, regions and countries who are committed to bringing air quality to safe levels by 2030. The initiative links partners together and supports action through providing a range of tools and sharing experience.	Primarily multiple actors and decision-makers in municipalities.	Worldwide relevance in helping maintain municipalities' focus on achieving better urban air quality.	snowball

Record built from citation database, snowball or peer recommendation.	In sourcebook?	Sourcebook Ref	Type of resource	Key entry point(s)	Author or Publisher	Date	Description	Audience (text as in sourcebook, otherwise blank)	Good for these situations (text as in sourcebook, otherwise blank)	Search Id
Food and Agriculture Organization (FAO). Social network analysis for territorial assessment and mapping of food security and nutrition systems (FSNS): a methodological approach. 2018. http://www.fao.org/3/I8751EN/i8751en.pdf	Y	32	Self audit	Food security and healthier nutrition	Food and Agriculture Organization (FAO)	2018	This work embodies a territorial approach to food security and nutrition policy, but also has strong links to the wider determinants of health and sustainability. This work is part of a broader effort of the FAO to support countries to improve the inclusiveness and sustainability of food security and nutritional systems. It aims to contribute to work on food systems and nutrition indicators, city-region food systems and rural-urban linkages.	All actors and decision-makers needing to understand and influence the inclusiveness, governance and efficiency of food systems from a food security and nutrition point of view.	Globally applicable methodological approach to analyse the social, institutional and economic dimensions of food systems and their relationships with food security and nutrition outcomes, as well as to assess the spatial patterns of food systems.	snowball
Food and Agriculture Organization (FAO). City region food systems programme. http://www.fao.org/in-action/food-for-citiesprogramme/approach/need-for-sustainableand-resilient-crfs/en/	Y	33	Network	Food security and healthier nutrition	Food and Agriculture Organization (FAO)	undated	A suite of online guidance, tools and information that offers concrete policy and programme opportunities through which rural and urban areas and communities in a given city-region can be directly linked. Directly addresses the wider determinants of health and sustainability through a territorial approach. Assessment and improvement of city-region food systems to help achieve better economic, social and environmental conditions in both urban and nearby rural areas.	Local governments in any country, including public health practitioners and local food activists.	The programme provides assistance in identifying and understanding gaps, bottlenecks and opportunities for sustainable planning, informed decision-making, prioritizing investments, designing sustainable food policies and strategies to improve local food systems.	snowball
WHO. Interventions on diet and physical activity: what works: summary report. 2009. https://www.who.int/dietphysicalactivity/whatworks/en/	Y	34	Evidence	Food security and healthier nutrition	WHO	2009	A summary of tried and tested diet and physical activity interventions that aim to reduce the risk of chronic NCDs. In terms of UTP, interventions in the following categories are included: policy, environment, workplace, schools, mass media, the community, primary health care, older adults and religious settings.	Policy-makers and stakeholders.	Public health promotion – diet and physical activity interventions to reduce the risk of chronic NCDs. Outlines interventions that use existing community social structures, such as schools or weekly meetings of older adults.	peer

Record built from citation database, snowball or peer recommendation.	In sourcebook?	Sourcebook Ref	Type of resource	Key entry point(s)	Author or Publisher	Date	Description	Audience (text as in sourcebook, otherwise blank)	Good for these situations (text as in sourcebook, otherwise blank)	Search Id
WHO. Health Equity Assessment Toolkit. https://www.who.int/gho/health_equity/assessment_toolkit/en/	Y	35	Analytical tool	Social and environmental justice	WHO	undated	The toolkit is a software application that facilitates the assessment of within country health inequalities. It can be used on desktops, laptop computers and mobile devices. It enables users to explore inequality in a setting of interest (e.g. a country, province or district) to determine the latest situation of inequality and the change in inequalities over time. It also allows users to compare inequality in the setting of interest with other settings.	Public health practitioners and spatial planners.	Assessing inequalities using disaggregated data and summary measures and advocacy through visualizing results via a variety of interactive graphs, maps and tables.	peer
Global Land Tool Network with UN-Habitat. Global Land Tool Network. https://gltn.net/	Y	36	Web resource	social justice; environmental justice; rights; land; tenure;	Global Land Tool Network with UN-Habitat	undated	The Global Land Tool Network is an alliance of international partners committed to increasing access to land and tenure security for all, with a particular focus on the poor and women. It uses a rights-based approach. The network's partners include international civil society organizations, research and training institutions, bilateral and multilateral organizations, and international professional bodies.	LMICs and any rapidly expanding city with land-rights issues.	A suite of land rights-based tools covering a range of subject areas.	peer
Block by Block https://www.blockbyblock.org/about	Y	37	Design guide	participation;	Block by Block	undated	Block by Block began in 2012 with the idea of integrating the computer game Minecraft into public space planning to get community members more involved. The approach is easy to use, and people of all ages, backgrounds and education levels can pick it up quickly.	Community and neighbourhood residents, including children and youth. Using a videogame to collect data and do planning charettes motivates children and youth to get involved.	LMICs; an effective, and cost-effective way to visualize a three dimensional environment, in a format designed for rapid iteration and idea sharing and advocacy. Helps neighbourhood residents model their surroundings, visualize possibilities, express ideas, drive consensus and accelerate progress	peer

Record built from citation database, snowball or peer recommendation.	In sourcebook?	Sourcebook Ref	Type of resource	Key entry point(s)	Author or Publisher	Date	Description	Audience (text as in sourcebook, otherwise blank)	Good for these situations (text as in sourcebook, otherwise blank)	Search Id
Inclusive Healthy Places: A Guide to Inclusion & Health in Public Space: Learning Globally to Transform Locally. Gehl Institute 2018 Web resources at https://gehlinstitute.org/wp-content/uploads/2018/07/Inclusive-Healthy-Places_Gehl-Institute.pdf	Y	38	Design guide	equity; inclusion;	Gehl Institute	2018	A participatory tool for evaluating and creating inclusive, healthy public places that support health equity. This framework supports inclusion to advance health equity through public spaces. The framework is built around four guiding principles for shaping and assessing public space projects. Only one principle addresses physical space, reflecting the need for practitioners to look beyond physical design and placemaking to create change. The process considers context, process and sustainability. The framework allows users to adapt and apply the approach to their situation in different ways.	Community, business and public sector. Mixed groups of actors and decision-makers including professionals with communities who are vulnerable and often marginalized.	Adaptable to a very wide applicability and relevant to different situations for improvement of public space towards inclusion and health.	snowball
World Health Organization & WHO Centre for Health Development (Kobe, Japan). (2010). Urban HEART: Urban Health Equity Assessment and Response Tool. World Health Organization http://www.who.int/iris/handle/10665/79060	Y	39	Toolkit	equity; inclusion;	WHO & WHO Centre for Health Development, Kobe, Japan	2010	The tool guides users through a standardized procedure of gathering relevant evidence and planning efficiently for appropriate actions to tackle health inequities. Case studies demonstrate how it has galvanized both city governments and communities to recognize and take action on health inequities.	Local policy-makers and communities. It is envisaged that cities in varied contexts can locally adapt and institutionalize the process, while maintaining its core concepts and principles.	It is designed for ease of use and to link evidence to action.	WHO S1
WHO. Don't pollute my future! The impact of the environment on children's health. 2017. https://www.who.int/ceh/publications/don-t-pollute-my-future/en/	Y	40	Briefing	children; child-friendly	WHO	2017	Broad and evidence-based briefing on environmental risk in childhood, focusing on specific diseases; it concludes with the information that reducing environmental risks could prevent a quarter of childhood deaths and disease. It also provides a review of the SDGs in relation to childhood risk and disease.	Public health practitioners.	Particularly good review of communicable disease risk to children in built environments.	Peer
Shaping urbanization for children. A handbook on child-responsive urban planning Aerts, Jens, (2018), United Nations Children's Fund (UNICEF), New York. Available from: https://www.unicef.org/publications/index_103349.html	Y	41	Design guide	children; child-friendly	UNICEF	2018	This handbook on child-responsive urban planning provides details for creating thriving and equitable cities where children live in healthy, safe, inclusive, green and prosperous communities. By focusing on children, this publication provides guidance on the central role that urban planning should play in achieving the SDGs.	All those accountable in the urban planning process, including city officials, real estate industry leaders, community leaders and planning, transport and public health practitioners.	Applicable for global perspectives and local contexts for all cities. It provides a highly accessible presentation of concepts, evidence and technical strategies to bring children to the foreground of urban planning.	snowball

Record built from citation database, snowball or peer recommendation.	In sourcebook?	Sourcebook Ref	Type of resource	Key entry point(s)	Author or Publisher	Date	Description	Audience (text as in sourcebook, otherwise blank)	Good for these situations (text as in sourcebook, otherwise blank)	Search Id
Cities Alive: Designing for urban childhoods. Arup. London 2107. Available at https://www.arup.com/perspectives/cities-alive-urban-childhood	Y	42	Design guide; briefing	children; child-friendly	ARUP	2017	This report highlights why and how city stakeholders should start to create child-friendly urban environments. It begins by highlighting the changing urban context, then sets out five core challenges of urban childhoods: traffic and pollution; high-rise living and urban sprawl; crime, social fears and risk aversion; isolation and intolerance; and inadequate and unequal access to the city.	All those accountable or having an interest in the urban planning process.	The report explores the benefits that child-friendly practices can bring, illustrated by case studies from around the world.	snowball
Measuring the age-friendliness of cities, WHO 2015. http://apps.who.int/iris/bitstream/handle/10665/203830/9789241509695_eng.pdf	Y	43	Toolkit	age-friendly; elders	WHO	2015	The tool is based on the perspectives and inputs of older people, care givers and service providers collected in 33 cities across all six WHO regions: Africa, Americas, Eastern Mediterranean, Europe, South-East Asia and Western Pacific. The publication focuses on eight key domains of urban life that encompass determinants of health and well-being: outdoor spaces and buildings; transportation; housing; respect and social inclusion; civic participation and employment; social participation; community and health services; and communication and information.	Public health practitioners and age-friendly communities of interest.	Providing a baseline for the promotion of age-friendly urban policies in cities worldwide.	WHO S1
Creating age-friendly environments in Europe, WHO and EU, 2016. Available from: http://www.euro.who.int/__data/assets/pdf_ file/0011/359543/AFEE-handbook.PDF	Y	44	Toolkit	age-friendly; elders	WHO Regional Office for Europe	2016	A handbook based on lessons learned from existing age-friendly initiatives in Europe. It builds on relevant locally and regionally developed tools that are now available, with evidence from research. The handbook links actions to create more age-friendly environments to the broader context of European health and social policies for ageing populations. There is a focus is on the interconnectedness and synergies between eight domains and how they can work together to address common goals such as increasing social inclusion, fostering physical activity or supporting people living with dementia.	Multisectoral partners in local governments and communities who work with them.	Demonstrates how local governments can create age-friendly environments.	peer
WHO. WHO Global Network for Age-friendly Cities and Communities. https://www.who.int/ageing/projects/age_friendly_cities_network/en/	Y	45	Network	age-friendly; elders	WHO	undated	The network was established to foster the exchange of experience and mutual learning between cities and communities worldwide.	Multisectoral partners in local governments and communities who work with them. Cities and communities in the network are of different sizes and are located in different parts of the world.	Support for acting on the desire and commitment to promote healthy and active ageing and a good quality of life for older residents.	peer

Record built from citation database, snowball or peer recommendation.	In sourcebook?	Sourcebook Ref	Type of resource	Key entry point(s)	Author or Publisher	Date	Description	Audience (text as in sourcebook, otherwise blank)	Good for these situations (text as in sourcebook, otherwise blank)	Search Id
WHO. WHO Housing and health guidelines. 2018. https://www.who.int/sustainable-development/publications/housing-health-guidelines/en/	Y	46	Evidence	housing	WHO	2018	Covers key areas of housing such as crowding, indoor temperature, accessibility, home injuries and summarizes other relevant WHO guidelines. The guidelines encompass general considerations for policy and good practice recommendations for addressing health problems.	Purposely designed for a broad audience in both the developed and developing world. The main target audience is policy-makers responsible for housing-related policies and regulations, enforcement measures.	Reducing risk factors, while recognizing the importance of key interventions. Initiating intersectoral collaboration that seeks to support healthy housing from a government perspective.	peer
A Practical Guide to Designing, Planning, and Executing Citywide Slum Upgrading Programmes. UN-Habitat, 2014 https://unhabitat.org/books/a-practical-guide-to-designing-planning-and-executing-citywide-slum-upgrading-programmes/	Y	47	Toolkit	slums; informal setttlements	UN-Habitat	2018	This guide for citywide slum upgrading and local projects provides a comprehensive manual with tips and tools from practical experience. It is an accessible tool for practitioners, leading them through UN-Habitat steps towards a successful citywide slum-upgrading programme.	Actors and decision-makers, including communities, public health and planners, involved with informal settlements and slums.	Accessible quick guide section provides an important reference tool for practitioners to help address the most pressing problems and the most important considerations in slum upgrading.	WHO_Slum
GENTRIFICATION & NEIGHBORHOOD CHANGE Helpful Tools for Communities. 2015. Voorhees, N. P., University of Chicago http://voorheescenter.red.uic.edu/wp-content/uploads/sites/122/2017/10/Gentrification-and-Neighborhood-Change-Toolkit.pdf	Y	48	Toolkit	equity;	NP Voorhees, University of Illinois at Chicago	2017	This toolkit argues that it is possible to have development without displacement and shows how to achieve this. Published as a supplement to The socioeconomic change of Chicago's community areas (1970–2010), the toolkit presents strategies for addressing the pressures of gentrification in a community during different phases of gentrification: before it happens, as it is happening, and after a neighbourhood has been gentrified.	A starting point for municipal collaboration with community residents, non-profit organizations, local businesses, elected officials and developers.	Helping to ensure that gentrification does not lead to population displacement, many of the tools and lessons could be widely applicable outside the USA.	snowball
UN-Habitat. City Prosperity Initiative. https://www.buildhealthyplaces.org/content/uploads/2018/04/Gentrification-and-Neighborhood-Change-Toolkit.pdf	Y	49	Web resource	local economy	UN-Habitat	undated	UN-Habitat's City Prosperity Initiative is a global initiative that has been applied in over 400 cities across the world. It provides an innovative approach to urban measurements and assists decision-makers to design clear policy interventions. In terms of health, some of the categories, such as equity and inclusion, and the ability to compare across cities could be of use.	Decision-makers in cities covered by this programme and mayors and city leaders wanting to join the programme.	Overview of some of the higher level indicators that are relevant to healthy UTP.	peer

Record built from citation database, snowball or peer recommendation.	In sourcebook?	Sourcebook Ref	Type of resource	Key entry point(s)	Author or Publisher	Date	Description	Audience (text as in sourcebook, otherwise blank)	Good for these situations (text as in sourcebook, otherwise blank)	Search Id
Health in the green economy: health co-benefits of climate change mitigation - transport sector. World Health Organization 2012. http://www.who.int/iris/handle/10665/70913	Y	50	Briefing	local economy	WHO	2012	This document is part of WHO's Health in the green economy series. It describes how many climate change measures can be "win-wins" for people and the planet. These policies can yield large, immediate public health benefits while reducing the upward trajectory of greenhouse gas emissions. The document is very comprehensive with case studies, and applicable to all countries. As well as plenty of background material, it outlines health benefits of transport-related greenhouse gas reduction strategies.	Widely applicable globally for city and national policy-makers across climate, transport, planning and public health sectors.	Assessing, planning and financing healthy transport interventions.	WHO S1
UN-Habitat. Participatory budgeting. https://pb.unhabitat.org/	Y	51	Briefing	local economy	UN-Habitat	undated	The tool uses technology to include citizens in the decision-making process for their city's budget. The benefits derive from the tool's short-term results and concrete outcomes for those involved. For example, participatory health budgeting can lead to prioritization of and investment in public health issues addressing citizens' real needs.	National governments, local authorities.	Identify common interests and concerns and linking people for joint action in public health, transparency in public health expenditure, encouraging accountability and responsibility of politicians.	peer
GIZ & WHO. Urban transport and health. Module 5g. Sustainable transport: a sourcebook for policy-makers in developing cities. 2011. https://www.who.int/hia/green_economy/giz_transport.pdf?ua=1	Y	52	Training	transport	GIZ and WHO	2011	A training module and sourcebook for practical orientation, focusing on best practices in planning and regulation with examples of successful experiences in developing cities. It provides an overview of the key pathways by which transport can influence health, and the scale of transport-related health risks in OECD and developing countries. It then discusses instruments that are available to assess and counter transport-related health risks.	Policy-makers in developing cities in transport, planning and public health sectors.	Offering some principles that can be used to guide the development of healthy transport systems. The sourcebook can be printed and provided to officials involved in urban transport. It can be easily adapted to fit a formal shortcourse training event on urban transport.	snowball

Record built from citation database, snowball or peer recommendation.	In sourcebook?	Sourcebook Ref	Type of resource	Key entry point(s)	Author or Publisher	Date	Description	Audience (text as in sourcebook, otherwise blank)	Good for these situations (text as in sourcebook, otherwise blank)	Search Id
Urban Land Institute, Building Healthy Corridors: Transforming Urban and Suburban Arterials into Thriving Places, 2016. Available from: https://uli.org/wp-content/uploads/ULI-Documents/Building-Healthy-Corridors-ULI.pdf	Y	53	Design Guide	transport; streets	Urban Land Institute	2017	This guide explores strategies for transforming commercial corridors (through roads dominated by commercial premises on each side), into places that support the health of the people who live, work and travel along them. This report is the result of a 2-year project that involved partnerships with four communities in the USA that are working to improve a specific corridor in ways that positively affect health. This report serves as a resource and reference for those who are undertaking corridor redevelopment efforts. It highlights the importance of health in decision-making processes; and it provides guidance, strategies and insights for reworking corridors in health-promoting ways.	Local businesses, communities and urban regeneration interests and actors.	Although the examples come from the USA, the principles and many lessons will be applicable in other high and middle-income countries in lowdensity urban contexts.	ULI
GIZ. Urban mobility plans: national approaches and local practice. Moving towards strategic, sustainable and inclusive urban transport planning. Sustainable urban transport technical document #13. 2014. http://www.transferproject.org/wp-content/uploads/2017/09/Urban-Mobility-Plans.pdf	Y	54	Overview	transport	GIZ	2014	Urban mobility plans are used as a planning tool and policy instrument to guide the development of transport in urban areas and surroundings. This document reviews urban mobility planning from several countries, showing a shift away from the traditional, infrastructure-oriented approach towards sustainable and people-oriented planning. National guidelines for urban mobility planning provide orientation to local authorities. In several countries, such as Brazil, France and India, the development of urban mobility plans has become an obligatory requirement for receiving national government funds for local transport projects to promote health.	Local policy-makers and planners who want to shape urban mobility processes and policies in an effective and inclusive manner. Policy-makers and experts at national level shaping state-of-the art national policy frameworks for urban transport planning.	Worldwide applicability for supporting low-carbon and active travel.	snowball
World Health Organization Regional Office for Europe. Health in impact assessments: opportunities not to be missed. Eds: Fehr, R., Viliani, F. Nowacki, J. and Martuzzi, M. 2014. http://www.euro.who.int/__data/assets/pdf_file/0011/261929/Health-in-Impact-Assessments-final-version.pdf?ua=1	Y	55	Overview	health impact assesments; HIA;	WHO Regional Office for Europe	2014	This publication aims to provide a detailed view on HIAs. Five key types of impact assessment, namely environmental impact assessment, strategic environmental assessment, social impact assessment, sustainability assessment, and HIA, are presented, and key questions are discussed. How can the various assessments contribute to promoting and protecting human health? How can further integration of health support the various forms of impact assessments?	Policy-makers and researchers.	Gaining a broad understanding at the potential for impact assessments to better protect and promote health.	peer

Record built from citation database, snowball or peer recommendation.	In sourcebook?	Sourcebook Ref	Type of resource	Key entry point(s)	Author or Publisher	Date	Description	Audience (text as in sourcebook, otherwise blank)	Good for these situations (text as in sourcebook, otherwise blank)	Search Id
WHO. Health impact assessment. https://www.who.int/hia/en/	Y	56	Web resource	health impact assesments; HIA;	WHO	undated	Main site and repository of information from the WHO about HIA.	For a range of environmental and health policy-makers worldwide.	Background, resources and examples on HIAs.	peer
UN-Habitat (in development). UN-Habitat health focused planning system assessment. unhabitat-ig-utp@un.org	Y	57	Training	health impact assesments; HIA;	UN-Habitat	undated	The assessment is a brief healthy planning assessment for national planning systems. This has been run as a workshop by UN-Habitat and is still in development. As a participatory workshop it allows participants to start to gain an overview of their planning system in terms of the degree to which it might be supportive of health outcomes.	Planners and public health practitioners and policy-makers.	Scoping the strengths and weaknesses of a planning system with reference to how it supports population health.	peer
Health Impact Project & the American Planning Association. Health impact assessment can inform planning to promote public health. 2016. https://planning-org-uploaded-media.s3.amazonaws.com/document/Health-Impact-Assessment-Can-Inform.pdf	Y	58	Briefing	health impact assesments; HIA;	Health Impact Project & the American Planning Association	2016	This brief introduces planning directors and staff as well as policy-makers to HIA, a process that brings public health considerations into decision-making. It describes how HIAs can add value across a range of topics and summarizes the findings from a review of 134 planning-related HIAs conducted in the USA between 2004 and 2014.	Planners and public health practitioners.	Explaining the role of HIA in planning with examples.	snowball
Centre for Urban Design and Mental Health. Measuring mental health outcomes in built environment research: choosing the right screening assessment tools. https://www.urbandesignmentalhealth.com/uploads/1/1/4/0/1140302/mental_health_assessment_tools_for_built_environment_research.pdf	Y	59	Analytical tool	health impact assesments; HIA;	Centre for Urban Design and Mental Health	undated	The centre curates and creates research and dialogue to inspire, motivate and empower policy-makers and urban practitioners to build mental health into their projects for a healthier, happier urban future.	Policy-makers, architects, transport planners, urban planners, developers, designers, engineers, geographers, and others who want to design better mental health into cities.	Design decision in relation to mental health.	snowball

Record built from citation database, snowball or peer recommendation.	In sourcebook?	Sourcebook Ref	Type of resource	Key entry point(s)	Author or Publisher	Date	Description	Audience (text as in sourcebook, otherwise blank)	Good for these situations (text as in sourcebook, otherwise blank)	Search Id
Propensity to Cycle project. Propensity to Cycle Tool. http://www.pct.bike/	Y	60	Web resource	cumulative exposures and risks	Propensity to Cycle project	undated	The Propensity to Cycle project was designed to assist transport planners and policy-makers to prioritize investments and interventions to promote cycling. It answers the question, "where is cycling currently common and where does cycling have the greatest potential to grow?" The tool can be used at different scales; all data so far and the project itself is based in England and Wales.	Transport professionals and researchers seeking new methodologies to support the promotion of cycling interventions.	Decision support in promoting cycling-based investments and policies.	snowball
Technical Centre for Disaster Risk Management, Sustainability and Urban Resilience with UN-Habitat. City Resilience Action Planning Tool. http://dimsur.org/	Y	61	Training	cumulative exposures and risks	Technical Centre for Disaster Risk Management, Sustainability and Urban Resilience with UN-Habitat.	undated	Known as CityRAP, this tool is used for training technicians in small to intermediate sized cities in sub-Saharan Africa. CityRAP enables communities to understand and plan actions aimed at reducing risk and building resilience through the development of a resilience framework for action. It is designed as an enabling rather than prescriptive tool, as the core principle is fostering ownership by local government and communities. The tool's design allows local governments to adapt and implement it with minimal external intervention. It draws on participatory methods, such as local government self-assessments, participatory risk mapping exercises and cross-sectorial action planning, to leverage local knowledge for understanding and planning resilience.	City managers and municipal technicians in local governments and urban stakeholders.	The tool includes a set of trainings, exercises and activities directed at municipalities that want to kick-start their resilience action planning.	peer
University of Melbourne. Low carbon living co-benefits calculator. https://thud.msd.unimelb.edu.au/tools-andmodels/co-benefits-calculator	Y	62	Analytical tool	cumulative exposures and risks	University of Melbourne	undated	The aim of the project is to develop and trial a prototype low-carbon precinct co-benefits calculator for use by urban planners and designers. The calculator estimates co-benefits associated with a range of alternative precinct designs and transport/land-use configurations across health, productivity and pollution associated with greenhouse gases and particulate emissions. The calculator will estimate population health status (with respect to chronic disease and injury) and productivity at a precinct (or greater) level.	Government regulators, developers, precinct planners, designers and local government officials.	Estimate the population health and productivity effects of various precinct design scenarios.	snowball
WHO. GreenUr: green space and urban planning tool. https://www.who.int/sustainable-development/urban/guidance-tools/en/	Y	63	Analytical tool	green spaces	WHO	undated	GreenUr calculates the impact of urban green spaces on health exposure, including cardiovascular disease. GreenUr is a flexible geographic information system plugin.			peer

Record built from citation database, snowball or peer recommendation.	In sourcebook?	Sourcebook Ref	Type of resource	Key entry point(s)	Author or Publisher	Date	Description	Audience (text as in sourcebook, otherwise blank)	Good for these situations (text as in sourcebook, otherwise blank)	Search Id
AirQ+: software tool for health risk assessment of air pollution http://www.euro.who.int/en/health-topics/environment-and-health/air-quality/activities/airq-software-tool-for-health-risk-assessment-of-air-pollution	Y	64	Analytical tool	air quality	WHO	undated	AirQ+ performs calculations that allow quantification of the health effects of exposure to air pollution, including estimates of the reduction in life expectancy. It can estimate the effects of short-term changes in air pollution and the effects of long-term exposures.	Public health professionals working in or with cities. The tool is designed for use in Europe although other areas may find it useful to review the methods used.	AirQ+ can be used for cities, countries or regions to estimate how much of a particular health effect is attributable to selected air pollutants compared to the current scenario; and what would be the change in health effects if air pollution levels changed in the future?	peer

Record built from citation database, snowball or peer recommendation.	In sourcebook?	Sourcebook Ref	Type of resource	Key entry point(s)	Author or Publisher	Date	Description	Audience (text as in sourcebook, otherwise blank)	Good for these situations (text as in sourcebook, otherwise blank)	Search Id
Health Economic Assessment Tool (HEAT) http://www.euro.who.int/en/health-topics/environment-and-health/Transport-and-health/activities/guidance-and-tools/health-economic-assessment-tool-heat-for-cycling-and-walking	Y	65	Analytical tool	transport	WHO	undated	Estimates the value of reduced mortality that results from regular cycling or walking. This is intended to be part of comprehensive cost–benefit analyses of transport interventions or infrastructure projects. It is based on best available evidence, with parameters that can be adapted to fit specific situations. However, its default parameters are valid for the European context. The tool calculates the answer to the following question: if x people cycle or walk y distance on most days, what is the economic value of mortality rate improvements?	Transport and public health professionals working in or with cities.	Wide range of uses, including planning a new piece of cycling or walking infrastructure: it models the impact of different levels of cycling or walking, and attaches a value to the estimated level when the new infrastructure is in place; or to value the mortality benefits from current levels of cycling or walking, such as benefits from cycling or walking to a specific workplace, across a city or in a country; or to estimate the mortality benefits from achieving national targets to increase cycling or walking, or to illustrate potential cost consequences of a decline in current levels of cycling or walking. This is largely validated for European cities, however, other cities will find the methodology useful and may be able to adapt the tool using their own data.	snowball
The Health Impact Project's cross-sector toolkit for health. https://www.pewtrusts.org/en/projects/health-impact-project	Y	66	Toolkit	health impact assessment;	Pew Charitable Trusts with Robert Wood Johnson Foundation	undated	A wealth of resources and toolkit to promote healthier communities through cross-sector collaboration. The cross-sector toolkit for health contains resources that help communities, agencies and other organizations take action to improve public health. The toolkit offers a collection of HIAs, guides and other research to support policy-makers' efforts to consider health when making decisions across sectors, such as housing, planning and education.	Civil society and multidisciplinary teams.	Health impact in planning projects in the USA.	snowball

Record built from citation database, snowball or peer recommendation.	In sourcebook?	Sourcebook Ref	Type of resource	Key entry point(s)	Author or Publisher	Date	Description	Audience (text as in sourcebook, otherwise blank)	Good for these situations (text as in sourcebook, otherwise blank)	Search Id
WHO. WHO global air pollution platform and database. https://www.who.int/health-topics/air-pollution	Y	67	web resource	spatial epidemiology; air quality	WHO	undated	Key information and monitoring source for cities on ambient and household air pollution.	Cities worldwide.	Access to a wide range of urban and rural resources to support healthier air quality.	peer
Slum Dwellers International. SDI Know Your City: community-driven data on slums. http://knowyourcity.info/	Y	68	Web resource	citizens science; slums	Slum Dwellers International	undated	This tool has been developed by Slum Dwellers International, a network of community-based organizations of the urban poor in 32 countries and hundreds of cities and towns across Africa, Asia and Latin America. Know Your City is a global network of knowledge that is owned by the communities it serves and has become the basis of a platform that supports an informed and united voice of the urban poor. It is becoming one of the largest repositories of informal settlement data in the world.	Anyone needed to share or access urban in Africa, Asia and Latin America including researchers, policy-makers, local governments and national governments.	Informal settlement data.	peer
Hush City. Hush City mobile phone application. http://www.opensourcesoundscapes.org/hush-city/	Y	69	Analytical tool	citizen science	Hush City	undated	Hush City is a mobile phone application that can be used by citizens to analyse and construct a local sound map with decibel levels and photos to identify, access and evaluate "everyday quiet areas" in neighbourhoods. It can be a useful tool to bring people together to collaborate in baseline mapping and exploring the role of sound, which has implications for urban stress and wellbeing.	Civil society and multidisciplinary teams.	Citizen supported data sourcing and mapping sound levels in different locations and at different times with simultaneous qualitative user information survey and quantitative capture.	snowball
Health in the green economy : health co-benefits of climate change mitigation - housing sector, 2011, http://www.who.int/iris/handle/10665/44609	N		Overview, briefing, evidence	housing; co-benefits	WHO	2012	This document is part of WHO's Health in the green economy series. It describes how many climate change measures can be "win-wins" for people and the planet. These policies can yield large, immediate public health benefits while reducing the upward trajectory of greenhouse gas emissions. The document is very comprehensive with case studies, and applicable to all countries. As well as plenty of background material, it outlines health benefits of transport-related greenhouse gas reduction strategies.			WHO S1

Record built from citation database, snowball or peer recommendation.	In sourcebook?	Sourcebook Ref	Type of resource	Key entry point(s)	Author or Publisher	Date	Description	Audience (text as in sourcebook, otherwise blank)	Good for these situations (text as in sourcebook, otherwise blank)	Search Id
Take Action To Improve Health: Act on What's Important. Robert Wood Johnson Foundation. Undated http://www.countyhealthrankings.org/key-activities/18392#key-activity-1	N		Toolkit	data; health impact	Pew Charitable Trusts with Robert Wood Johnson Foundation	undated	A wealth of resources and toolkit to promote healthier communities through cross-sector collaboration. The cross-sector toolkit for health contains resources that help communities, agencies and other organizations take action to improve public health. The toolkit offers a collection of HIAs, guides and other research to support policy-makers' efforts to consider health when making decisions across sectors, such as housing, planning and education.			ULI
Housing: shared interests in health and development, 2011. Geneva : World Health Organization. http://www.who.int/iris/handle/10665/44705	N		Briefing	housing	WHO	2011	Millions of people struggle every day with poor housing, overcrowding, lack of affordability, and lack basic services connected to their homes. Poor transportation services between areas of home and work, and for social activities, often create barriers to accessing employment and social opportunities. These issues present challenges to housing policy-makers and have implications for the health and well-being of populations. As stated in the Adelaide Statement on Health in All Policies, "well-designed, accessible housing and adequate services can successfully address fundamental determinants of health for disadvantaged individuals and communities". For the housing sector, paying attention to the health impacts of its policies can yield huge benefits. Indeed, housing interventions that consider citizens' quality of life create better living conditions and improve housing policies' sustainability. For the health sector, improving determinants of health such as housing is key in reducing health-care costs. In England, poor housing, damp and mould, cold, overcrowding, fires, and domestic injuries as a result of falls on floors cost the National Health Service (NHS) up to £600 million a year.			WHO S1

Record built from citation database, snowball or peer recommendation.	In sourcebook?	Sourcebook Ref	Type of resource	Key entry point(s)	Author or Publisher	Date	Description	Audience (text as in sourcebook, otherwise blank)	Good for these situations (text as in sourcebook, otherwise blank)	Search Id
Transport (road transport) : shared interests in sustainable outcomes 2011 http://www.who.int/iris/handle/10665/44749	N		Briefing	co-benefits	WHO	2011	In depth report of co-benefits using transport as the entry point.<br>Goal 1. Sustainable transport systems enhance economic development, while minimizing potential negative impacts -Transport should support the efficient movement of people, goods and services to contribute to economic development and minimize the negative impacts associated in particular with congestion<br>Goal 2. Sustainable transport systems improve safety -Transport systems should be safe throughout the entire network, including roads, pedestrian zones and vehicles, and should be designed to avoid and reduce injuries and fatalities, and contribute to the health of local populations.<br>Goal 3. Sustainable transport systems ensure everyone can access transport services and facilities without barriers -Transport systems should be designed to serve the needs of all people, addressing the barriers that prevent mobility, especially for disadvantaged groups.<br>Goal 4. Transport systems promote environmentally sustainable transport options -Transport should ensure mobility by adopting environmentally sound systems and modes.<br>Goal 5. Sustainable transport systems promote mobility conducive to livelihood security and liveable communities - Transport systems should contribute to social cohesion by addressing congestion; improving public transport systems and policies aimed at reducing car use; developing infrastructure for pedestrians and cyclists; and by encouraging social interaction and livelihood security.			WHO S1

Record built from citation database, snowball or peer recommendation.	In sourcebook?	Sourcebook Ref	Type of resource	Key entry point(s)	Author or Publisher	Date	Description	Audience (text as in sourcebook, otherwise blank)	Good for these situations (text as in sourcebook, otherwise blank)	Search Id
Prüss-Ustün, A, J Wolf, C Corvalán, R Bos and M Neira. Preventing Disease Through Healthy Environments: A Global Assessment of the Burden of Disease from Environmental Risks. WHO; 2016. http://apps.who.int/iris/bitstream/handle/10665/204585/9789241565196_eng.pdf	N		Evidence	overview; advocacy	WHO	2016	This report presents a wide-ranging assessment and detailed findings to show by how much and in what ways improving the environment can promote health and well-being.<br>This study provides an approximate estimate of how much disease can be prevented by reducing the environmental risks to health. It includes a meta-synthesis of key evidence relating diseases and injuries to the environment. It brings together quantitative estimates of the disease burden attributable to the environment using a combination of approaches that includes CRA, epidemiological data, transmission pathways and expert opinion. The synthesis of evidence linking 133 diseases and injuries, or their groupings, to the environment has been reviewed to provide an overall picture of the disease burden that could be prevented through healthier environments. Environmental risks to health are defined, in this study, as "all the physical, chemical and biological factors external to a person, and all related behaviours, but excluding those natural environments that cannot reasonably be modified." To increase the policy relevance of this study, its focus is on that part of the environment which can reasonably be modified			WHO_Slum
Healthy Streets for London: Prioritising walking, cycling and public transport to create a healthy city. 2017.Transport for London. Available at http://content.tfl.gov.uk/healthy-streets-for-london.pdf	N		Initiative	high streets;	Transport for London	2017	The Healthy Streets Approach provides the framework of policies and strategies we will put in place to achieve this. At a street level, direct investment in our walking, cycling and public transport infrastructure is vital to providing a safer, easier, cleaner and more appealing environment for everyone to enjoy. At a network level, we must design and manage our streets and rail systems so that more active travel becomes part of every journey. And we need to plan for the future. As London continues to grow, active travel needs to be designed into the fabric of new developments and regeneration projects.			HUDU

Record built from citation database, snowball or peer recommendation.	In sourcebook?	Sourcebook Ref	Type of resource	Key entry point(s)	Author or Publisher	Date	Description	Audience (text as in sourcebook, otherwise blank)	Good for these situations (text as in sourcebook, otherwise blank)	Search Id
5 Keys to Healthier Cities. IS Global https://www.isglobal.org/en/ciudadesquequeremos	N		Web resource	identifying entry points; air pollution; noise; natural spaces; physical activity; climate; air quality;	IS Global	undated	Our health - and that of our planet - depends on how cities are designed. Urban life poses challenges that push us to rethink how we plan urban environments. The #CitiesWeWant are cities designed for people: places where we can live well and be healthy. In this interactive report, and over the next weeks, ISGlobal researchers will detail the 5 key strategies to building healthy and sustainable cities.<br>Good for prioritisation and identifying entry points.			snowball
Integrating Health Impact Assessment in Urban Design and Planning: The Manukau Experience A report prepared in 2010 by Adrian Field for the Ministry of Health. 2011. Ministry of Health, New Zealand. https://www.health.govt.nz/system/files/documents/publications/hia-urban-design-v2.pdf	N		Initiative	health impact assesments; HIA;	Ministry of Health, New Zealand	2010	Worked example of an HIA for an urban plan.			peer
Health as the pulse of the new urban agenda: United Nations conference on housing and sustainable urban development, Quito, October 2016 http://www.who.int/iris/handle/10665/250367	N		Overview	planning; new urban agernda; health protection; placemaking	WHO	2016	This report considers how to integrate health into urban planning, investments, and policy decisions, so as to support the implementation and achievement of the goals and objectives of the New Urban Agenda. Two core themes run throughout this report. First, to realize Habitat III's collective vision of sustainable, liveable and economically vibrant cities, urban decision-makers must apply a "health lens" – to fully assess the risks and opportunities posed by their policies and programs, and measure their effects. Second, achieving truly sustainable urban development will require much greater cross-sectoral coordination to protect and improve the health of vulnerable populations in the world's fast-growing urban areas.			WHO S1

Record built from citation database, snowball or peer recommendation.	In sourcebook?	Sourcebook Ref	Type of resource	Key entry point(s)	Author or Publisher	Date	Description	Audience (text as in sourcebook, otherwise blank)	Good for these situations (text as in sourcebook, otherwise blank)	Search Id
Regional framework for urban health in the Western Pacific 2016-2020: Healthy and resilient cities. 2016. http://iris.wpro.who.int/handle/10665.1/13047	N		Overview	cities;	WHO	2016	Moves Healthy Cities from a settings to systems approach. Acknowledges the role of built environment and the urban planned environment in health outcomes.			WHO S1
Global report on urban health: equitable healthier cities for sustainable development 2016 http://www.who.int/iris/handle/10665/204715	N		Evidence	overview; advocacy	WHO	2016	In 2010 we published our first joint global report on urban health titled 'Hidden cities: unmasking and overcoming health inequities in urban settings'. The global community waking up to this new demographic reality, the consequent implications for health, society, and economic and political affairs; and also to a host of new opportunities as humans shape and are shaped by an urban future. As well as impact of urbanization on human health, the report demonstrated that the growth of prosperity in cities leaves behind significant 'hidden' urban areas and populations. Many of today's urban poor are not only much worse off than their fellow citizens, they even lag behind rural populations. Urban inequity is obviously unjust, and hindered national and global achievement of the Millennium Development Goals. This new report coincides with the advent of the new SDGs and development paradigm. Equity, inclusiveness, and accountability in health and development are core principles and themes of the SDGs, as well as for the Third United Nations Conference on Housing and Sustainable Urban Development in Quito in October 2016. The commitment to universal health coverage, as well as to the New Urban Agenda that will emerge from Quito, is intrinsically linked to improving the living conditions and health of all city dwellers.			WHO S1

Record built from citation database, snowball or peer recommendation.	In sourcebook?	Sourcebook Ref	Type of resource	Key entry point(s)	Author or Publisher	Date	Description	Audience (text as in sourcebook, otherwise blank)	Good for these situations (text as in sourcebook, otherwise blank)	Search Id
Healthy cities : good health is good politics : toolkit for local governments to support healthy urban development 2015 http://iris.wpro.who.int/handle/10665.1/11865	N		Toolkit	advocacy; equity; HIA	Manila : WHO Regional Office for the Western Pacific	2015	This toolkit aims to highlight key considerations in applying the Healthy Cities approach and using it as a platform to achieve healthy urban development. It emphasizes key actions a city can take to protect and promote health. Each city should consider its local context and needs, and adopt what it thinks is best for the city. And throughout the implementation, the city should continuously strive to align actions to the core principles of Healthy Cities. This toolkit is not a guideline for addressing health challenges in a city and meeting specific standards. The resources and case studies are intended to serve as examples and are not gold standards for application. Assess health inequities and identify social determinants using the tools described in Chapter 4 – such as the Urban Health Equity Assessment and Response Tool (Urban HEART), Health Lens Analysis, Health Impact Assessment (HIA) or Rapid Equity-Focused Health Impact Assessment (Rapid EFHIA).			WHO S1
Health risk assessment of air pollution – general principles. Copenhagen: WHO Regional Office for Europe; 2016. https://www.euro.who.int/en/health-topics/environment-and-health/air-quality/publications/2016/health-risk-assessment-of-air-pollution.-general-principles-2016	N		Briefing	HIA; air pollution; air quality;	WHO EURO	2016	Important for UTP are the document helps look at the policy implications of the assessment. An air pollution health risk assessment (AP-HRA) estimates the health impact to be expected from measures that affect air quality, in different socioeconomic, environmental, and policy circumstances. As such, it is an important tool for informing public policy decisions. This document introduces the concept of AP-HRA, describes in broad terms how the health risks of outdoor air pollution and its sources are estimated, and gives an overview of the general principles for the proper conduct of an AP-HRA for various scenarios and purposes. The information is aimed at a broad audience of readers who do not need to know how to apply the tools, but seek a general understanding of the concepts, scope and principles of AP-HRA.			peer

Record built from citation database, snowball or peer recommendation.	In sourcebook?	Sourcebook Ref	Type of resource	Key entry point(s)	Author or Publisher	Date	Description	Audience (text as in sourcebook, otherwise blank)	Good for these situations (text as in sourcebook, otherwise blank)	Search Id
The Evolution of National Urban Policies A Global Overview 2014 by UN-Habitat Nairobi https://unhabitat.org/the-evolution-of-national-urban-policies	N		Overview	land use; spatial planning; planning policy; growth; intensification;	UN-Habitat	2014	Background: The high-level lessons emerging are as follows:<br>1 - Active support needs to be mobilized across the different spheres of government to ensure a coordinated approach to planning and managing cities and towns.<br>2 - Implementation requires a sustained technical process to develop the legal foundations, capable institutions and financial instruments to design and build more productive, liveable and resilient cities and towns.<br>3 - Effective delivery requires active collaboration between spheres of government along with the devolution of appropriate responsibilities and resources.<br>4 - An important objective is to manage the peripheral expansion of cities in the interests of more compact and inclusive urban growth.			snowball
Aboelata, M.J., Bennett, R., Yañez, E, Bonilla, A., & Akhavan, N. (2017). Healthy Development Without Displacement: Realizing the Vision of Healthy Communities for All. Oakland, CA: Prevention Institute. Available at https://www.buildhealthyplaces.org/content/uploads/2018/04/Healthy-Development-without-Displacement-realizing-the-vision-of-healthy-communities-for-all.pdf	N		Briefing	equity; gentrification; regeneration	Prevention Institute	2017	This paper:<br>1 - Underscores the health impacts of displacement, and how community stability supports public health<br>2 - Highlights some of the organizations, researchers, and communities leading this work, and key publications that dive deeper into these issues<br>3 - Promotes new alliances between those working on healthy community initiatives and those working deeply on anti-displacement and affordable housing<br>4 - Amplifies the call for shared action across sectors and issue areas Aim to spark further discussion, collaboration, and innovation in the fiel.d			snowball

Record built from citation database, snowball or peer recommendation.	In sourcebook?	Sourcebook Ref	Type of resource	Key entry point(s)	Author or Publisher	Date	Description	Audience (text as in sourcebook, otherwise blank)	Good for these situations (text as in sourcebook, otherwise blank)	Search Id
United Nations University (2015). Principles for Healthy and Sustainable Places. Kuching. UNU-IIGH. Dr. Patrick, Deakin University. https://i.unu.edu/media/iigh.unu.edu/news/4079/Principles-for-Healthy-and-Sustainable-Places.pdf	N		Overview	inspiration; engagement; participation; leadership	United Nations University	2015	Overivew and advocacy. Ten inspiring principles: Human and ecosystem health are centre stage in good place design. Physical activity, mental health, social connection and healthy eating are fostered through natural and built environments that are designed to connect, respect and protect.			snowball
Investing in water and sanitation: increasing access, reducing inequalities: GLAAS 2014 findings - highlights for the Region of the Americas 2016 http://www.who.int/iris/handle/10665/204597	N		Briefing	water; sanitation; WASH;	WHO	2016	Good example of pan-regional monitoring and assessment report.<br>Sixteen countries out of 35 in the WHO Americas Region, total population of 550 million, participated in the GLAAS 2013/2014 reporting cycle. Overall, access to improved drinking-water and sanitation services in the Region of the Americas is 96 and 87 per cent (in 2015), respectively. More than 110 million people gained access to an improved drinking-water source and over 400 million gained access to improved sanitation in the 2005 to 2015 time period. However, there were still over 100 million people without improved sanitation and over 35 million without access to an improved drinking-water source in the Region of the Americas.<br>Progress has been made on water and sanitation in the region. However, there is still a substantial need to further strengthen government commitments and actions to approve and implement national policies and plans for the provision of safe and sustainable water and sanitation services. Challenges include:<br>• Reducing inequalities in access to water and sanitation,<br>• Applying the human right to water and sanitation to ensure access of services to all,<br>• Building capacity for surveillance of water supplies,<br>• Creating action plans to fill the gap in human resources, and<br>• Establishing a comprehensive national system for planning and implementing WASH sector financing.			WHO S1

Record built from citation database, snowball or peer recommendation.	In sourcebook?	Sourcebook Ref	Type of resource	Key entry point(s)	Author or Publisher	Date	Description	Audience (text as in sourcebook, otherwise blank)	Good for these situations (text as in sourcebook, otherwise blank)	Search Id
Urbanization and health: health equity and vulnerable populations: case studies from the Eastern Mediterranean Region. 2010. http://www.who.int/iris/handle/10665/119914	N		Initiative	intersectoral cooperation and collaboration	WHO EMRO	2010	The preliminary evidence presented by these studies provides a powerful argument for city planners, municipal authorities, nongovernmental organizations and civil society in their efforts to improve urban health. It is intended to be used as an advocacy tool to sensitize policy-makers and parliamentarians to urban health issues and influence them to make positive changes. It underscores the need for intersectoral collaborative action by governments, health development partners, United Nations agencies, nongovernmental organizations, medical professionals and civil society.			WHO S1
Addressing the social determinants of health: the urban dimension and the role of local government. WHO Europe 2012. https://apps.who.int/iris/handle/10665/327956	N		Evidence	cities, leadership; equity;	WHO EURO	2012	This report summarizes the evidence on the social determinants of health and the built environment with special reference to the role of local government across countries in the WHO European Region. It draws on the findings of the global Commission on Social Determinants of Health and the European review of social determinants of health and the health divide. Through its leadership, local government has a significant role to play in working across sectors and with civil society partners to support and accelerate action to address the social determinants of health and the causes of health inequalities. The evidence presented here provides the background to the complementary report Healthy cities tackle the social determinants of inequities in health: a framework for action.			peer
The Human Rights in Cities Handbook Series: Volume I: The Human Rights-Based Approach to Housing and Slum Upgrading. 2017. Nairobi. UN-Habitat. Availabale online at https://unhabitat.org/books/the-human-rights-in-cities-handbook-series-volume-i-the-human-rights-based-approach-to-housing-and-slum-upgrading/	N		Initiative	slums; informal settlements;	UN-Habitat	2017	The Handbook also advocates for the use of the Human Rights Based Approach in preventive measures (such as the provision of new housing stock and policy) as well as curative interventions (such as the upgrading of slums and informal settlements) in the planning and design, implementation, and the monitoring and evaluation phases. As such, it prioritizes re-focussing urban development priorities to those in vulnerable situations to ensure that they benefit from and are involved in strategies or combatting inequalities and segregation in towns and cities.			snowball

Record built from citation database, snowball or peer recommendation.	In sourcebook?	Sourcebook Ref	Type of resource	Key entry point(s)	Author or Publisher	Date	Description	Audience (text as in sourcebook, otherwise blank)	Good for these situations (text as in sourcebook, otherwise blank)	Search Id
CLARK, P., MAPES, N., BURT, J. & PRESTON, S. 2013. Greening Dementia - a literature review of the benefits and barriers facing individuals living with dementia in accessing the natural environment and local greenspace. Natural England Commissioned Reports, Number 137. [NECR137_edition_1]	N		Evidence	older people; elders; age-friendly	Natural England	2013	Evidence suggests that social interaction and access to the outdoors and nature is important for people living with dementia and that these activities have an important role in their quality of life.<br>2. Evidence of benefits for people living with dementia from access to the natural environment is limited and often qualitative. However, general findings can be grouped into:<br>• Improved emotional state: reduced stress, agitation, anger, apathy and depression<br>• Improved physical health: skin health, fitness, sleeping patterns, eating patterns<br>• Improved verbal expression<br>• Improved memory and attention<br>• Improved awareness: multi-sensory engagement and joy<br>• Improved sense of well-being, independence, self-esteem and control<br>• Improved social interaction and a sense of belonging			snowball
Floater, G., Rode, P., Friedel, B. and Robert, A., 2014. Steering urban growth: Governance, policy and finance. NCE Cities – Paper 02. LSE: London.	N		Briefing	land use; spatial;	Floater, G., Rode, P., Friedel, B. and Robert, A	2014	Introduced the 3C model of urban development:<br>Pillar 1: Compact urban growth<br>Pillar 2: Connected infrastructure<br>Pillar 3: Coordinated governance			snowball
Linking urban transport and land use in developing countries. Robert Cervero. Journal of Transport and Land Use, Vol. 6, No. 1 (2013), pp. 7-24 https://conservancy.umn.edu/handle/11299/171183	N		Evidence	land use; spatial; transport; equity	Robert Cervero.	2013	Call for integrated transport and land-use planning needs to be elevated in importance in developing cities before it is too late.<br><br>Examples reviewed in this paper from South Asia, Southeast Asia, China, India, Africa, and South America. It is concluded that whatever is done to improve transportation and land-use integration must be pro-poor.The cardinal features of integrated and sustainable transport and urbanism everywhere—accessible urban activities and safe, attractive walking and cycling environs—are particularly vital to the welfare and prosperity of urbanites in the world's poorest countries.			snowball

Record built from citation database, snowball or peer recommendation.	In sourcebook?	Sourcebook Ref	Type of resource	Key entry point(s)	Author or Publisher	Date	Description	Audience (text as in sourcebook, otherwise blank)	Good for these situations (text as in sourcebook, otherwise blank)	Search Id
Settlement patterns, urban form and sustainability: An evidence review. RTPI 2018 https://www.rtpi.org.uk/research/2018/may/settlement-patterns-urban-form-and-sustainability/	N		Evidence	land use; spatial;	RTPI	2018	Chapters on the relationship between settlement patterns, urban form and economic productivity, Climate change, Public health, Ageing population.			snowball
The Case for Healthy Places: Improving health outcomes through placemaking, Project for Public Spaces, 2016. https://daks2k3a4ib2z.cloudfront.net/5810e16fbe876cec6bcbd86e/5a626855e27c0000017efc24_Healthy-Places-PPS.pdf	N		Toolkit	place-making; participation	Project for Public Spaces	2016	Both the quality and process of placemaking, defined loosely within this publication as a project that occurs in public spaces helping people to feel connected to a place and greater community, has demonstrated physical, mental, and social health benefits.<br><br>KEY TAKEAWAYS:<br>Both the quality and process of placemaking, defined loosely within this publication as a project that occurs in public spaces helping people to feel connected to a place and greater community, has demonstrated physical, mental, and social health benefits.			snowball
Health Indicators of sustainable cities in the Context of the Rio+20 UN Conference on Sustainable Development. Initial findings from a WHO Expert Consultation: 17-18 May 2012 [indicators_cities] http://www.who.int/hia/green_economy/indicators_cities.pdf?ua=1 WHO/HSE/PHE/7.6.2012f	N		Briefing	health Indicators; sustainable development;	WHO	2012	Health indicators proposed here also reflect progress on social equity, environment , and development dimensions of sustainable cities.<br>Core indicators include:<br>§ Slum housing improvements that benefit health – as assessed by well-defined measures for safe, resilient, and climate-adapted structures that also have access to clean energy and basic utilities;<br>§ Urban air quality in terms of particulate pollution – with respect to WHO air quality guidelines;<br>§ Healthy, efficient transport – in terms of safety and use of sustainable modes, including walking, cycling, and public transport;<br>§ Urban violence - in terms of intentional homicides.<br>Governance indicators assess how cities account for health in urban planning and building codes, and in monitoring of air/water quality and sanitation risks.<br>Indicators of access to urban services essential to public health and sustainable cities also are			peer

Record built from citation database, snowball or peer recommendation.	In sourcebook?	Sourcebook Ref	Type of resource	Key entry point(s)	Author or Publisher	Date	Description	Audience (text as in sourcebook, otherwise blank)	Good for these situations (text as in sourcebook, otherwise blank)	Search Id
Urban health equity assessment and response tool project [Urban HEART]: socioeconomic determinants and health status of people living in Sale City, Morocco 2012 http://www.who.int/iris/handle/10665/116819	N		Initiative	health equity; city assessment	World Health Organization. Regional Office for the Eastern Mediterranean	2012	Actions taken outside the health sector are likely to have greater effect than those taken through the health sector alone. Corrective action must include changes in the social determinants of health, such as the elimination of slums, creation of industrial parks, development of income- generating activities and the assurance of education for all. The Urban HEART approach will enable decision-makers to become informed about the priorities of their area by providing easily read colour-coded results to help them put health at the centre of attention. Morocco is experiencing political change and it would be advisable to integrate the Government's global strategy regarding social determinants of health into regions to enable the Urban HEART approach to be fully integrated into the health programme of each region.' P29			WHO S1
Can urban regeneration improve health resilience in a changing climate? Lessons from Indonesia. Rukuh Setiadi, Johanna Nalau. WoRKING PAPER SERIES 23: 2015. Asian Cities Climate Resilience. Book/Report, 46 pages [File:Setiadi and Nalau 2017 10744IIED]	N		Initiative	urban regeneration; climate	Asian Cities Climate Resilience	2015	Solid background and foundation lessons for practitioners * Assessment of the extent that urban regeneration, in particular housing for low-income groups, is being considered as an adaptation option in Indonesian cities * Exploration of the potential challenges and outcomes in using urban regeneration, particularly housing construction for low-income groups, as an adaptation strategy for enhancing health resilience in particular, at the city level in Indonesia.			IIED
Mainstreaming environment and climate change: Health G03139 Published: Jul 2011 - IIED and Irish Aid Briefing	N		Briefing	poverty; climate; environment	IIED and Irish Aid	2011	Robust briefing on climate change and health covering vestor-bourne diseases, poor water, sanitation and hygiene, chemical pollutants, rising urban populations, degradation of natural resources.			IIED

Record built from citation database, snowball or peer recommendation.	In sourcebook?	Sourcebook Ref	Type of resource	Key entry point(s)	Author or Publisher	Date	Description	Audience (text as in sourcebook, otherwise blank)	Good for these situations (text as in sourcebook, otherwise blank)	Search Id
URBAN PLANNING AND DESIGN LABS: tools for integrated and participatory urban planning. Quito, Habitat III Version 1.0 UN-Habitat 2016<br><br>https://unhabitat.org/programme/urban-lab	N		Initiative	participation; design proposals;	UN-Habitat	2016	Urban planning is a complex process that requires strong political leadership and commitment, and implementable legal frameworks articulated through a common development vision and social participation. It is exactly in the complexity of this process that UN-Habitat founded its in-house Urban Planning and Design Lab, in order to provide assistance to local, regional and national authorities with urban planning and design. The UN-Habitat´s Lab has been a response to a growing demand from cities, not only providing tools for their urbanization challenges, but also responding to urban planning in general. Within a complex set of actors and technical realities, the Planning Lab finds its existence and application to create, navigate and accelerate the urban strategies and transformative projects for implementation. This publication aims to present the Lab´s approach towards planning and design, management and implementation, as well as to introduce the trajectory of the UN-Habitat's Lab through its working years of experience.			snowball
Towards More Physical Activity in Cities: Transforming public spaces to promote physical activity — a key contributor to achieving the Sustainable Development Goals in Europe WHO and EU 2017. Available at http://www.euro.who.int/__data/assets/pdf_file/0018/353043/2017_WHO_Report_FINAL_WEB.pdf	N		Briefing	public space; physical activity	WHO	2017	This publication focuses on physical activity and how it can be supported through urban planning.<br><br>The focus on physical activity is explained by the fact that inactivity today accounts for an increasing proportion of deaths and disability worldwide and is associated with significant health care costs and productivity losses.2 Action to increase rates of physical activity will be necessary to achieve global targets on the prevention of premature mortality from noncommunicable diseases – the leading cause of death worldwide – and to halt the rise in obesity. With more than 80% of the European population expected to live in urban areas by 2030, cities play a pivotal role in promoting and protecting health and well-being.3 As cities continue to expand in population, there is a growing need to develop ways of supporting physical activity in dense urban settings.			snowball

Record built from citation database, snowball or peer recommendation.	In sourcebook?	Sourcebook Ref	Type of resource	Key entry point(s)	Author or Publisher	Date	Description	Audience (text as in sourcebook, otherwise blank)	Good for these situations (text as in sourcebook, otherwise blank)	Search Id
Healthy Environments for Healthier People WHO EURO 2018. http://www.euro.who.int/__data/assets/pdf_file/0006/367188/eceh-eng.pdf	N		Briefing	health protection ; transport; air ;	WHO EURO	2018	Good as a reference on health protection.			Peer
Providing health intelligence to meet local needs: a practical guide to serving local and urban communities through public health observatories. 2014. WHO Centre for Health Development (Kobe, Japan) http://www.who.int/iris/handle/10665/152645	N		Briefing	public health observatories	WHO Centre for Health Development (Kobe, Japan)	2014	This practical guide makes the case for the use of local public health observatorie. It goes through key considerations for developing one, as well as ways to ensure effective functioning. It includes successful case studies of the London Health Observatory and the City of Juárez Observatory.			WHO S1
Guiding Principles for City Climate Action Planning UN-Habitat 2015	N		Design guide	climate	UN-Habitat	2015	Sets out guiding principles for climate action planning on the city-level, along with guiding steps for the planning process and case examples. Fairly comprehensive and sets out examples related to both health and UTP, but is not focused on either. Health - Air quality improvement measures including reductions of short lived climate pollutants; heat wave (or cold snap) health action plans; prevention of spread of diseases affected by climate change.			snowball
Urban Planning for City Leaders UN-Habitat 2013	N		Briefing	Political leadership ;	UN-Habitat	2013	Comprehensive briefing on urban planning for local authorities / mayors, looking at UTP to accomplish various goals. None specific to health, although health co-benefits are addressed.			snowball
Urban HEART : urban health equity assessment and response tool: user manual. 2010. http://www.who.int/iris/handle/10665/79061	N		Toolkit	health equity	WHO Kobe	2010	User manual for step by step use of Urban Heart assessment method			WHO S1

Record built from citation database, snowball or peer recommendation.	In sourcebook?	Sourcebook Ref	Type of resource	Key entry point(s)	Author or Publisher	Date	Description	Audience (text as in sourcebook, otherwise blank)	Good for these situations (text as in sourcebook, otherwise blank)	Search Id
Urban Design Guidelines to Mitigate Urban Heat Island (UHI) Effects In Hot-Dry Cities. By: Taslim, Shima; Parapari, Danial Monsefi; Shafaghat, Arezou JURNAL TEKNOLOGI Volume: 74 Issue: 4 Special Issue: SI Pages: 119-124 Published: 2015. Available at http://eprints.utm.my/id/eprint/55735/1/ArezouShafaghat2015_UrbanDesignGuidlinestoMitigateUrbanHeatIsland.pdf	N		Evidence	heat islands, climate;	Taslima, S., Paraparib, D.M. and Shafaghata, A.	2015	Academic paper with practical urban design guidelines setting out setting out recommendation for UTP, specifically street design, to mitigate urban heat islands. Mixed-methodology.			WoS
Planning the unplanned: incorporating agriculture as an urban land use into the Dar es Salaam master plan and beyond. Afton Halloran & Jakob Magid. Environment and urbanization, 2013, Vol.25(2), p.541-558. Available at https://www.researchgate.net/profile/Afton_Halloran/publication/262948915_Planning_the_unplanned_Incorporating_agriculture_as_an_urban_land_use_into_the_Dar_es_Salaam_master_plan_and_beyond/links/56a8f83b08ae7f592f0d5511/Planning-the-unplanned-Incorporating-agriculture-as-an-urban-land-use-into-the-Dar-es-Salaam-master-plan-and-beyond.pdf	N		Evidence; Overview	Food systems; urban agriculture	Halloran & Magid	2013	Academic paper looking at urban agriculture in Dar es Salaam, specifically it's lack of inclusion in formal urban development plans and attempts to legitimize the practice. Mixed methodology, but mainly based on fieldwork, participatory action research and stakeholder interviews. Specific to Dar es Salaam, but might also be interesting in other cities with a large degree of informal urban agriculture.			WoS

Record built from citation database, snowball or peer recommendation.	In sourcebook?	Sourcebook Ref	Type of resource	Key entry point(s)	Author or Publisher	Date	Description	Audience (text as in sourcebook, otherwise blank)	Good for these situations (text as in sourcebook, otherwise blank)	Search Id
Urban health equity assessment and response tool project [Urban HEART]: socioeconomic determinants and health status of people living Gezirat El Warak, Giza, Egypt 2012. http://www.who.int/iris/handle/10665/116820	N		Self audit	social determinants of health; equity	WHO	2012	Assessment of social determinants of health and health outcomes in Gezirat El Warak, Egypt, using the Urban HEART tool.			WHO S1
Urban Planning and Health Inequities: Looking in a Small-Scale in a City of Cape Verde Gonçalves. L, Santos Z, Amado M, Alves D, Simões R, et al. (2015) Urban Planning and Health Inequities: Looking in a Small-Scale in a City of Cape Verde. PLOS ONE 10(11): e0142955. Https://doi.org/10.1371/journal.pone.0142955	N		Evidence	equity; informal settlements	Gonçalves, L., Santos, Z., Amado, M., Alves, D., Simões, R., Delgado, A.P., Correia, A., Cabral, J., Lapão, L.V. and Craveiro, I.	2015	Academic paper looking at health disparities between urban residents in Praia, Cape Verde living in various urban units - formal, transition and informal. Considers multiple health outcomes as well as residents own perception. The lack of high-quality data to support evidence-based policies continues to be a concern in African cities, which present marked social, economic and cultural disparities that may differently impact the health of the groups living in different urban contexts. This study explores three urban units—formal, transition and informal—of the capital of Cape Verde, in terms of overweight/obesity, cardiometabolic risk, physical activity and other aspects related to the urban environment.			WoS
Warren Smit, Ariane de Lannoy, Robert V.H. Dover, Estelle V. Lambert, Naomi Levitt, Vanessa Watson. Making unhealthy places: The built environment and non-communicable diseases in Khayelitsha, Cape Town. Health & Place,Volume 39, 2016,Pages 196-203,ISSN 1353-8292. https://doi.org/10.1016/j.healthplace.2016.04.006. (http://www.sciencedirect.com/science/article/pii/S1353829216300247)	N		Evidence	NCDs; urban design	Smit, W., de Lannoy, A., Dover, R.V., Lambert, E.V., Levitt, N. and Watson, V	2016	Academic paper analysing how the built environment in Khayelitsha, Cape Town contributes to the health of it's residents, focusing mainly on food, physical activity and mental health.			ACfC

Record built from citation database, snowball or peer recommendation.	In sourcebook?	Sourcebook Ref	Type of resource	Key entry point(s)	Author or Publisher	Date	Description	Audience (text as in sourcebook, otherwise blank)	Good for these situations (text as in sourcebook, otherwise blank)	Search Id
Prasad, A., Kano, M., Dagg, K.A.M., Mori, H., Senkoro, H.H., Ardakani, M.A., Elfeky, S., Good, S., Engelhardt, K., Ross, A. and Armada, F. Prioritizing action on health inequities in cities: an evaluation of urban health equity assessment and response tool (urban heart) in 15 cities from Asia and Africa. Social science & medicine, Volume 145, 2015, Pages 237-242, Https://doi.org/10.1016/j.socscimed.2015.09.031. (http://www.sciencedirect.com/science/article/pii/s0277953615301337	N		Self audit	equity;	Prasad, A., Kano, M., Dagg, K.A.M., Mori, H., Senkoro, H.H., Ardakani, M.A., Elfeky, S., Good, S., Engelhardt, K., Ross, A. and Armada, F	2015	Evaluation of a pilot project using Urban HEART in 15 cities, looking at how closely cities had followed the recommendations of the tool, identified barriers and facilitators in the process of using the tool and main suggestions for improving it by pilot cities.			WoS
The East Asian age-friendly cities promotion – Taiwan's experience and the need for an oriental paradigm. Tzuyuan Stessa Chao, Huiwen Huang. Global Health Promotion.Vol 23, Issue 1_suppl, pp. 85 – 89. First Published May 19, 2016. https://doi-org.proxy.kib.ki.se/10.1177/1757975916641612	N		Briefing; Initiative	age-friendly; elders	Chao, T.S. and Huang, H.	2016	Report on Taiwan's experience using the WHO handbook 'Global Age-friendly Cities (AFC): A Guide'. Argues the need for an East Asian-specific approach in implementing the handbook due to specific cultural aspects around ageing. This paper identifies three key elements for AFC promotion in East Asian countries based on an analysis of Taiwan's experience: during needs assessment take collectivism into consideration, during action plans at the community level community leaders' views will be more important (particularism), and when promoting AFC at the institutional level a top-down approach will be more acceptable (high power distance concept)			PubMed_S1

Record built from citation database, snowball or peer recommendation.	In sourcebook?	Sourcebook Ref	Type of resource	Key entry point(s)	Author or Publisher	Date	Description	Audience (text as in sourcebook, otherwise blank)	Good for these situations (text as in sourcebook, otherwise blank)	Search Id
Turley R, Saith R, Bhan N, Rehfuess E, Carter B. Slum upgrading strategies involving physical environment and infrastructure interventions and their effects on health and socio-economic outcomes. Cochrane Database of Systematic Reviews 2013, Issue 1. Art. No.: CD010067. https://doi.org/10.1002/14651858.CD010067	N		Evidence	slums	Turley, R., Saith, R., Bhan, N., Rehfuess, E. and Carter, B.	2013	Systematic review on slum upgrading interventions in the physical environment or infrastructure improvements, assessing the impact health, quality of life and socio-economic wellbeing of LMIC urban slum dwellers. Comprehensive review of slum upgrading programmes across low and middle income countries, collating and synthesizing interventions to provide a broad picture of their impacts for different settings, interventions and outcomes. It also considers and exposes key methodological issues in undertaking systematic reviews of these sorts of complex interventions.			PubMed_S3
Tiwari, G. (2014), "Planning and Designing Transport Systems to Ensure Safe Travel for Women", International Transport Forum Discussion Papers, No. 2014/04, OECD Publishing, Paris. http://dx.doi.org/10.1787/5jz40rjgtjxx-en	N		Briefing	gender; transport; equity	Tiwari, G.	2014	Discussion paper on planning transport systems for safe travel, specifically looking at differences in travel patterns between men and women and ensuring safe travel for women. Examples mainly from India but recommendations are more general.			OECD
NHS London Healthy Urban Development Unit, Healthy Urban Planning Checklist, 2017. Available from: https://www.healthyurbandevelopment.nhs.uk/wp-content/uploads/2017/05/Healthy-Urban-Planning-Checklist-3rd-edition-April-2017.pdf	N		Toolkit	planning policy;	NHS London Healthy Urban Development Unit	2017	Checklist for healthy urban development, specifically to ensure new developments are in line with London policy requirements and standards.			HUDU
Urban Land Institute Los Angeles, 'Healthy Community Implementation Toolbox', 2016. Available from: https://ulidigitalmarketing.blob.core.windows.net/ulidcnc/sites/47/2020/04/ULI-Healthy-Community-Implementation-Toolbox-FINAL.pdf	N		Overview; Toolkit; Urban design	legislation; equity; urban design;	ULI	2016	Overview of how various policy tools (ie zoning codes, design guidelines, building codes etc.) can be used to improve community health. Specific to Los Angeles but applicable in the more general context too.			ULI

Record built from citation database, snowball or peer recommendation.	In sourcebook?	Sourcebook Ref	Type of resource	Key entry point(s)	Author or Publisher	Date	Description	Audience (text as in sourcebook, otherwise blank)	Good for these situations (text as in sourcebook, otherwise blank)	Search Id
Building with Nature. https://www.buildingwithnature.org.uk	N		Initiative; Design guide; Web Resource	green infrastructure	Building with nature	undated	UK initiative aiming to encourage green infrastructure and wildlife protection through standards for new developments. Comes with a user guide that aims to support developers and policy makers. Gives out accreditation to new developments that align with these voluntary standards. Has wellbeing as one of four themes within the framework.			snowball
Inclusive Design for Getting Outdoors. IDGO studies. https://www.idgo.ac.uk/about_idgo/index.htm	N		Web resource	age-friendly; elders; physical activity	IDGO group	undated	UK research initiative looking at the quality of life impact for older people of spending time outdoors, and the barriers and facilitators to that. Found higher quality of life and activity levels for participants who lived in environments that provided easier and more enjoyable outdoor activity and identified features that supported this. Practical evidence based factsheets and guidance.			snowball
Urban green spaces and health - a review of evidence WHO EURO. 2016. http://www.euro.who.int/__data/assets/pdf_file/0005/321971/Urban-green-spaces-and-health-review-evidence.pdf	N		Evidence	green space; evidence	WHO EURO	2016	This report summarizes the available evidence of beneficial effects of urban green spaces, such as improved mental health, reduced cardiovascular morbidity and mortality, obesity and risk of type 2 diabetes, and improved pregnancy outcomes. Mechanisms leading to these health benefits include psychological relaxation and stress alleviation, increased physical activity, reduced exposure to air pollutants, noise and excess heat. Characteristics of urban green spaces that are associated with specific mechanisms leading to health benefits, and measures or indicators of green space availability, accessibility and use that have been used in previous surveys are discussed from the perspective of their public health relevance and applicability for monitoring progress towards goals set in international commitments, such as the Parma Declaration in the WHO European Region and the global Sustainable Development Goals. The report also presents a suggested indicator of accessibility of green spaces with examples of its application in three European cities and a detailed methodological tool kit for GIS analysis of land use and population data.			snowball

Record built from citation database, snowball or peer recommendation.	In sourcebook?	Sourcebook Ref	Type of resource	Key entry point(s)	Author or Publisher	Date	Description	Audience (text as in sourcebook, otherwise blank)	Good for these situations (text as in sourcebook, otherwise blank)	Search Id
The Urban Health Index: A handbook for its calculation and use. Kobe, Japan: World Health Organization; 2014. http://www.who.int/iris/handle/10665/136839	N		Initiative	indicators; metrics	WHO	2014	n depth guide to statistical calculations for Urban Health Index and the reasoning behind them. Subtitled 'A VERSATILE APPROACH TO MEASURING AND MAPPING HEALTH INEQUALITIES'. The UHI provides a flexible approach to selection, amalgamation, and presentation of health data. Its purpose is to furnish visual, graphical, and statistical insight into various health indicators and health determinants within particular geographic boundaries and health disparities with a focus on capturing intra-urban health disparities. The UHI may be used by public health workers, evaluators, statisticians, program managers, academic researchers, and decision makers to examine the current status of urban areas, to assess change and the effect of program interventions, and to plan for urban improvements.			WHO S1
City leadership for health and sustainable development: Critical issues for successful Healthy Cities projects. 2017. Tsouros, A. [Tsouros - 2017 - City leadership for health and sustainable development Critical issues for successful Healthy Cities projects- annotated]	N		Overview	leadership	Tsourous, A.	2017	1. Introduction<br>2. An open letter to Mayors and Governors<br>3. Healthy Cities – The vision, values and goals of a project that became a global movement<br>4. What approaches Healthy Cities need to implement to be successful<br>5. Working across sectors: health in all policies and whole of government and whole of society approaches<br>6. Political leadership: the role of local governments, building consensus and strengthening commitment for health and well-being<br>7. Setting up Healthy Cities projects: Managing change and promoting innovation<br>8. A minimum action agenda for every modern Healthy City<br>9. Making it happen: preconditions for achieving better outcomes			snowball

Record built from citation database, snowball or peer recommendation.	In sourcebook?	Sourcebook Ref	Type of resource	Key entry point(s)	Author or Publisher	Date	Description	Audience (text as in sourcebook, otherwise blank)	Good for these situations (text as in sourcebook, otherwise blank)	Search Id
Nieuwenhuijsen, M. J. Urban and transport planning, environmental exposures and health-new concepts, methods and tools to improve health in cities. Environmental Health. 2016;15(Suppl 1):38. doi:10.1186/s12940-016-0108-1	N		Overview	health protection ; transport; air quality;	Nieuwenhuijsen, M. J.	2016	Cities have long been known to be society's predominant engine of innovation and wealth creation, yet they are also a main source of pollution and disease. We conducted a review around the topic urban and transport planning, environmental exposures and health and describe the findings. Within cities there is considerable variation in the levels of environmental exposures such as air pollution, noise, temperature and green space. Emerging evidence suggests that urban and transport planning indicators such as road network, distance to major roads, and traffic density, household density, industry and natural and green space explain a large proportion of the variability. Personal behaviour including mobility adds further variability to personal exposures.<br>Making cities 'green and healthy' goes far beyond simply reducing CO2 emissions. Environmental factors are highly modifiable, and environmental interventions at the community level, such as urban and transport planning, have been shown to be promising and more cost effective than interventions at the individual level. However, the urban environment is a complex interlinked system. Decision-makers need not only better data on the complexity of factors in environmental and developmental processes affecting human health, but also enhanced understanding of the linkages to be able to know at which level to target their actions. New research tools and assessment approaches can now provide this information.			PubMed_S1
UNESCO. PRIMED—A Successful Experience in Urban Intervention. UNESCO; 1996; available at http://unesdoc.unesco.org/images/0012/001297/129776eo.pdf	N		Overview;	case study; healthy cities; equity	UNESCO	1996	Fascinating story of early Urban and Territorial Planning work carried out in Medllin, Colombia.			WHO_Slum

Record built from citation database, snowball or peer recommendation.	In sourcebook?	Sourcebook Ref	Type of resource	Key entry point(s)	Author or Publisher	Date	Description	Audience (text as in sourcebook, otherwise blank)	Good for these situations (text as in sourcebook, otherwise blank)	Search Id
The economic impact of floods and waterlogging on low-income households: lessons from Indore, India<br>Pritee Sharma, Anup Karanth, Megha Burvey, Amit Dubey. Working paper, 35 pages. 2016.	N		Briefing	economy; flooding	Sharma, P., Karath, A., Burvey, M. and Dubey, A.	2016	This study estimates the economic losses suffered by the urban poor in terms of assets and productivity due to climate-induced waterlogging and floods. It examines how the vulnerability of slum dwellers living in informal settlements is exacerbated by a lack of supportive institutional mechanisms, the nature of non-inclusive economic growth, the social exclusion of urban landscapes and discriminative access to public services.			IIED
Rydin Y, Bleahu A, Davies M, et al. Shaping cities for health: complexity and the planning of urban environments in the 21st century. Lancet. https://www.ncbi.nlm.nih.gov/pmc/articles/PMC3428861/	N		Overview, evidence base,	advocacy;	Rydin et al.	2012	The Healthy Cities movement has been in process for almost 30 years, and the features needed to transform a city into a healthy one are becoming increasingly understood. What is less well understood, however, is how to deliver the potential health benefits and how to ensure that they reach all citizens in urban areas across the world. Complexity thinking stresses that the development of a plan that anticipates all future change for these issues will not be possible. Instead, incremental attempts to reach a goal need to be tried and tested. Such thinking suggests a new approach to planning for urban health—one with three main components.<br><br>First, there needs to be an emphasis on the promotion of experimentation through diverse projects and the use of trial and error to increase the understanding of how best to improve urban health outcomes in specific contexts. Second, this emphasis on learning from projects in turn suggests the need for strengthened assessment. However, a different kind of assessment is needed to that usually used for public health interventions. Third, consideration of the value-laden nature of policy interventions and the creation of forums to debate the moral and ethical dimensions of different approaches to urban health and city environments are essential.			PubMed_S1

Record built from citation database, snowball or peer recommendation.	In sourcebook?	Sourcebook Ref	Type of resource	Key entry point(s)	Author or Publisher	Date	Description	Audience (text as in sourcebook, otherwise blank)	Good for these situations (text as in sourcebook, otherwise blank)	Search Id
Durand CP, Andalib M, Dunton GF, Wolch J, Pentz MA. A Systematic Review of Built Environment Factors Related to Physical Activity and Obesity Risk: Implications for Smart Growth Urban Planning. Obesity reviews : an official journal of the International Association for the Study of Obesity. 2011;12(501):e173-e182. doi:10.1111/j.1467-789X.2010.00826.x	N		Evidence	obesity; Smart growth;	Durand et al.	2011	Five smart growth factors (diverse housing types, mixed land use, housing density, compact development patterns and levels of open space) were associated with increased levels of physical activity, primarily walking. Associations with other forms of physical activity were less common. Results varied by gender and method of environmental assessment. Body mass was largely unaffected. This review suggests that several features of the built environment associated with smart growth planning may promote important forms of physical activity. Future smart growth community planning could focus more directly on health, and future research should explore whether combinations or a critical mass of smart growth features is associated with better population health outcomes.			PubMed_S1
Healthy cities and the city planning process: a background document on links between health and urban planning. L.J. Duhl & A.K. Sanchez WHO Europe 1999	N		Overview	healthy cities;	WHO EURO	1999	Classic but a bit dated and overtaken by more accessible material			peer
Making the case for investment in the walking environment: A review of the evidence. Living Streets. https://www.livingstreets.org.uk/media/1394/2011-making-the-case-full-report.pdf	N		Evidence	walking; streets; economics;	Living Streets	2011	In depth look at nine different interventions types that can be made in the walking environment to assess a wide range of social, economic and health benefits using a wide evidence base. Ten case studies six from England and one each from New York, Melboune, Drachten and Copenhagen as used to illustrate good practice. Valuable and universally applicable conclusions about benefit to cost ratios and implementation.			snowball

Record built from citation database, snowball or peer recommendation.	In sourcebook?	Sourcebook Ref	Type of resource	Key entry point(s)	Author or Publisher	Date	Description	Audience (text as in sourcebook, otherwise blank)	Good for these situations (text as in sourcebook, otherwise blank)	Search Id
Place Standard Tool https://placestandard.scot	N		Web resource	place; place-making; participation;	NHS Scotland	n/a	The Place Standard tool provides a simple framework to structure conversations about place, whether the place is well-established, undergoing change, or is still being planned. It is a resource for participants to think about the physical elements of a place (for example its buildings, spaces, and transport links) as well as the social aspects (for example whether people feel they have a say in decision making). The tool provides prompts for discussions, allowing all the elements of a place to be considered in a methodical way. The tool pinpoints the assets of a place as well as areas where a place could improve. The website describes multiple applications for the tool in the place-making cycle.			peer
Pham, T. D. M., and T. T. S. Lam. "Gender needs and roles in building climate resilience in Hue City, Vietnam." Asian Cities Climate Resilience Network Working Paper Series 33 (2016). https://pubs.iied.org/pdfs/10780IIED.pdf	N		Initiative	climate; gender	Pham, T. D. M., and T. T. S. Lam	2016	This working paper examines gender roles in building climate resilience in Hue City. We conducted participatory research in 12 wards using the City Resilience Framework (Arup, 2014) to engage with local authorities, people and city planners. Hue City has its own special identity that significantly influences its resilience to climate change: health and well-being have been improved by investment in healthcare. In relation to gender roles, our study found that men and women at a grassroots level have different vulnerabilities and contribute differently to building climate resilience in Hue City. Women play key roles in sustaining and enhancing the health and well-being of people within their community, and accruing funds for households, communities and society. They also take part in organising mutual support for each other during times of disruption. By comparison, men are more engaged in activities relating to safety, security and other continuity plans within their communities. However, at the management level, we found that women hold only minor roles. There are therefore significant opportunities to challenge gender-based conceptions of capacity and responsibility, and to improve the gender sensitivity of decision-making processes and forums. This could significantly enhance the resilience of the people living in Hue City.			IIED

Record built from citation database, snowball or peer recommendation.	In sourcebook?	Sourcebook Ref	Type of resource	Key entry point(s)	Author or Publisher	Date	Description	Audience (text as in sourcebook, otherwise blank)	Good for these situations (text as in sourcebook, otherwise blank)	Search Id
Biophilic Public Health: Re-imagining Public Health for the 21st Century. Dr Neil de Wet. 2018. Toi Te Ora Public Health. https://www.toiteora.govt.nz/vdb/document/1973	N		Briefing	biophila; nature; biophilic design; trees; cities;	Toi Te Ora Public Health.	2018	Biophilic design helps make happy and productive workplaces; creates healing and comforting environments in hospitals; shapes schools where children thrive physically, emotionally and academically; and, supports well-being and community in homes and neighbourhoods. Biophilic buildings go well beyond 'eco-building' concepts of sustainable materials and energy efficiency, to design that nurtures and cares for people, community and nature, and in doing so expresses the values of love, care and respect for nature as well as, reciprocally, allowing nature to nurture our own health and well-being.			snowball
Building Better Cities with Young Children and Families. How to engage our youngest citizens and families in city building: a global scan of best practices. Bernard van Leer, Urban 95, 8-80 Cities 2017 https://bernardvanleer.org/app/uploads/2017/10/BvLF-8-80-Cities-Report-Final.pdf	N		Initiative	children; child-friendly;	Bernard van Leer	2017	This work began with extensive background research and in-depth interviews with leading practitioners in the field to discover existing resources and knowledge. We collected 21 case studies from 16 different countries and uncovered stories and ideas that demonstrate effective methods for engaging young children and caregivers in diverse aspects of city building.<br>We set out looking for cities and communities that are leading the charge when it comes to engaging young children and caregivers. We were unable to find any model cities where civic engagement is embedded across all departments and aspects of city life. The good news is that we found incredible stories of innovative programs or individual community leaders who are pushing for more inclusive approaches for city building.<br>This report is a starting point. The case studies and principles are meant to spark deeper thinking and meaningful dialogue about the importance of engaging these demographics. While the examples are inspiring, this report<br>is also a reminder that the status quo is not enough. City governments need to do more to integrate the needs of this often invisible but important group into decision-making processes.			peer

Record built from citation database, snowball or peer recommendation.	In sourcebook?	Sourcebook Ref	Type of resource	Key entry point(s)	Author or Publisher	Date	Description	Audience (text as in sourcebook, otherwise blank)	Good for these situations (text as in sourcebook, otherwise blank)	Search Id
Housing: Shared Interests in Health and Development, Social Determinants of Health, Sectoral Briefing Series 1. World Health Organization; 2011. Available at http://apps.who.int/iris/bitstream/handle/10665/44705/9789241502290_eng.pdf	N		Briefing	housing;	WHO	2011	The WHO Housing and health guidelines aim at informing housing policies and regulations at the national, regional and local level and are further relevant in the daily activities of implementing actors who are directly involved in the construction, maintenance and demolition of housing in ways that influence human health and safety. The guidelines therefore emphasize the importance of collaboration between the health and other sectors and joint efforts across all government levels to promote healthy housing. The guidelines' implementation at country-level will in particular contribute to the achievement of the Sustainable Development Goals on health (SDG 3) and sustainable cities (SDG 11). The policy recommendations provide a good outline and brefing but a more recent and in-depth evidence review is found in the WHO Housing and health guidelines guidelines, 2018 (Sourcebook Resource 46).			WHO_Slum
London Healthy Urban Development Unit, *Rapid Health Impact Assessment Tool*, 2019. Via https://www.healthyurbandevelopment.nhs.uk/our-services/delivering-healthy-urban-development/health-impact-assessment/	N		Toolkit	HIA;	London Healthy Urban Development Uni	2019	This rapid HIA tool is designed to assess the likely health impacts of development plans and proposals, including planning frameworks and masterplans for large areas, regeneration and estate renewal programmes and outline and detailed planning applications. It can be applied in a variety of processes, from desktop to community event. Although the introduction set the scene for Londin planning policy, as a resource, the supplied matix of impact assessment is widely applicable internationally. The tool provides an assessment matrix based on eleven topics or broad determinants: 1. Housing design and affordability, 2. Access to health and social care services and other social infrastructure, 3. Access to open space and nature, 4. Air quality, noise and neighbourhood amenity, 5. Accessibility and active travel, 6. Crime reduction and community safety, 7. Access to healthy food, 8. Access to work and training, 9. Social cohesion and inclusive design, 10. Minimising the use of resources, 11. Climate change.			HUDU

Record built from citation database, snowball or peer recommendation.	In sourcebook?	Sourcebook Ref	Type of resource	Key entry point(s)	Author or Publisher	Date	Description	Audience (text as in sourcebook, otherwise blank)	Good for these situations (text as in sourcebook, otherwise blank)	Search Id
Fredsgaard,M.W., Cave,B. and Bond,A. A review package for Health Impact Assessment reports of development projects. 2009. Ben Cave Associates Ltd. Available at https://www.scambs.gov.uk/media/5749/hia-review-package-ben-cave-assoc.pdf	N		Analytical tool	HIA; masterplans;	Ben Cave Associates Ltd	2009	This excellent review package contains a complete quality assessment process for HIAs of development projects. It focuses on assessing HIA reports prepared in the United Kingdom, however it also offered as the basis for other countries to modify the criteria in line with their own decision-making context and HIA practice. The review package treats the HIA report as a stand-alone document. In practice HIA is often conducted alongside, and may draw on the results of, other assessment studies. The results of the HIA may be presented as an integral part of a larger study or as a stand-alone report. The way in which the results are presented is likely to be decided by the commissioner of the report and other parties such as the planning authority and the responsible health authority.. We suggest that the coverage of health issues will still need to address the criteria specified in this review package.			snowball
Burgess, Katharine, et al. Harvesting the Value of Water: Stormwater, Green Infrastructure, and Real Estate. Washington, D.C.: Urban Land Institute, 2017.<br><br>https://uli.org/wp-content/uploads/ULI-Documents/HarvestingtheValueofWater.pdf	N		Briefing	water; flooding; economics; development	Urban Land Institute	2017	The report seeks to address a gap in today's research on stormwater management approaches. Although much has been written on the topic of green infrastructure and water management, most recent reports focus on stormwater policies or opportunities for capturing stormwater in the public realm. Fewer have focused on implications for private sector real estate developers. This report brings together an analysis of the stormwater policy landscape and an introduction to a variety of real estate development projects that have responded to them. After out-lining the reasons that stormwater management is important to cities, this report introduces a series of real estate case studies and a range of types of stormwater policies. The case studies come from locations across the United States and present both innovations in stormwater management and positive financial, operational, or design outcomes. US Case studies.			ULI

Record built from citation database, snowball or peer recommendation.	In sourcebook?	Sourcebook Ref	Type of resource	Key entry point(s)	Author or Publisher	Date	Description	Audience (text as in sourcebook, otherwise blank)	Good for these situations (text as in sourcebook, otherwise blank)	Search Id
Mark J. Nieuwenhuijsen, Haneen Khreis, Ersilia Verlinghieri, Natalie Mueller, David Rojas-Rueda. Participatory quantitative health impact assessment of urban and transport planning in cities: A review and research needs. Environment International. Volume 103. 2017. Pages 61-72. ISSN 0160-4120, https://doi.org/10.1016/j.envint.2017.03.022	N		Evidence	HIA; research;	ISGlobal	2017	An overview of the current state-of-the art of HIA in cities and made recommendations for further work. The process on how to get there is as important and will provide answers to many crucial questions on e.g. how different disciplines can effectively work together, how to incorporate citizen and stakeholder opinion into quantitative HIA modelling for urban and transport planning, how different modelling and measurement methods can be effectively integrated, and whether a public health approach can bring about positive changes in urban and transport planning.			PubMed_S1
Eitler, Thomas W., Edward T. McMahon, and Theodore C.Thoerig. Ten Principles for Building Healthy Places. Washington, D.C.: Urban Land Institute, 2013.<br><br>http://uli.org/wp-content/uploads/ULI-Documents/10-Principles-for-Building-Healthy-Places.pdf	N		Overview	cities; places; urban design; food; streets;	Urban Land Institute	2013	This publication explores the building of healthy places from the framing that physical design affects human behaviour at all scales—buildings, neighbourhoods, communities, and regions. The places in which we live, work, and play can affect both our mental and physical well-being. Today, communities across the United States are facing obesity and chronic disease rates of epic proportions. Our built environment offers both opportunities for and barriers to improving public health and increasing active living. Communities designed in a way that supports physical activity (wide sidewalks, safe bike lanes, attractive stairways, accessible recreation areas) encourage residents to make healthy choices and live healthy lives. Healthy places in turn create economic value by attracting both younger and older workers and appeal to a skilled workforce and innovative companies. US focus but applicable in all modern cities.			ULI

Record built from citation database, snowball or peer recommendation.	In sourcebook?	Sourcebook Ref	Type of resource	Key entry point(s)	Author or Publisher	Date	Description	Audience (text as in sourcebook, otherwise blank)	Good for these situations (text as in sourcebook, otherwise blank)	Search Id
Gender and Transport Resource Guide. Online Tool developed by the Sub-Saharan Africa Transport Program (SSATP), 2006. The World Bank.<br><br>https://www.ssatp.org/sites/ssatp/files/publications/HTML/Gender-RG/index.html	N		Web resource	gender; transport;	Sub-Saharan Africa Transport Program (SSATP) at The World Bank.	2006	This Resource Guide provides gender mainstreaming tools and information for individuals and groups working on policy, design, implementation monitoring and evaluation, capacity building and research in the transport sector and sectors affected by transport. The Resource Guide:<br>• Provides checklists, entry points, and tools for mainstreaming gender in transport projects and policies.<br>• Brings together case studies, best practices, training manuals, tools and reports on gender and transport from the Gender and Rural Transport Initiative (GRTI), and other initiatives of the World Bank and allied agencies.<br>• Uses a gender lens to analyse transport programs ranging from national infrastructure policies and plans to community driven development projects.<br>• Presents examples of engendering the full range of transport network interventions ranging from intermediate means of transport (IMT) such as bicycles, donkeys and carts to formal transport sub-sectors such as roads, buses and boats.<br>• Provides slide presentations that can be adapted for gender and transport training, gender sensitization, and other events.			snowball
Generating better evidence for sustainable development research and evaluation. IIED. https://www.iied.org/generating-better-evidence-for-sustainable-development-research-evaluation	N		Web resource	assets based approach; research; participation; informal settlements; slums; data collection;	IIED	2017	IIED is developing a body of work that seeks to understand how to develop better evidence for sustainable development research and evaluation. A group of IIED researchers and evaluators have explored how methodological innovations and time-tested approaches may contribute to better evidence for sustainable development, and developed a better evidence philosophy for sustainable development. This includes seven case studies of methodological approaches that can generate better evidence. Includes briefings covering several topic such as community-driven data collection in informal settlements and participatory resource mapping.			IIED

Record built from citation database, snowball or peer recommendation.	In sourcebook?	Sourcebook Ref	Type of resource	Key entry point(s)	Author or Publisher	Date	Description	Audience (text as in sourcebook, otherwise blank)	Good for these situations (text as in sourcebook, otherwise blank)	Search Id
World Health Organization. Keeping the vector out: housing improvements for vector control and sustainable development. World Health Organization; 2017. https://www.who.int/social_determinants/publications/keeping-the-vector-out/en/	N		Briefing	vector; housing;	WHO	2017	This policy brief "Keeping the vector out: housing improvements for vector control and sustainable development" contributes to recognize the importance of integrated approaches of vector control and aiming at highlighting effective housing interventions to prevent vector-borne diseases. The evidence shows that poor quality housing and neglected peri-domestic environments are risk factors for the transmission of malaria, arboviral diseases (e.g. dengue, yellow fever, chikungunya, Zika virus disease), Chagas disease and leishmaniasis and that housing interventions such as screening windows, doors and eaves of houses, by fitting ceilings, and by reducing the vectors' indoor hiding and breeding places, such as cracks and crevices in walls, floors and roofs are essential for reducing morbidity, mortality, human suffering and thereby promoting economic growth, well-being and the reduction of poverty.			WHO_Slum
Corburn, J. and Sverdlik, A., 2017. Slum upgrading and health equity. International journal of environmental research and public health, 14(4), p.342. doi:10.3390/ijerph14040342	N		Briefing	slums; informal settlements; health equity; development;	International journal of environmental research and public health	2017	Informal settlement upgrading is widely recognized for enhancing shelter and promoting economic development, yet its potential to improve health equity is usually overlooked. Almost one in seven people on the planet are expected to reside in urban informal settlements, or slums, by 2030. The processes and products of slum upgrading can address multiple environmental determinants of health. This paper reviewed urban slum upgrading evaluations from cities across Asia, Africa and Latin America and found that few captured the multiple health benefits of upgrading. We conclude with suggestions for how slum upgrading might more explicitly capture its health benefits, such as through the use of health impact assessment (HIA) and adopting an urban health in all policies (HiAP) framework. Urban slum upgrading must be more explicitly designed, implemented and evaluated to capture its multiple global environmental health benefits.			PubMed_S3

Record built from citation database, snowball or peer recommendation.	In sourcebook?	Sourcebook Ref	Type of resource	Key entry point(s)	Author or Publisher	Date	Description	Audience (text as in sourcebook, otherwise blank)	Good for these situations (text as in sourcebook, otherwise blank)	Search Id
Corburn, J., 2017. Urban place and health equity: critical issues and practices. International journal of environmental research and public health, 14(2), p.117. https://www.mdpi.com/1660-4601/14/2/117	N		Briefing	health equity; cities	International Journal of Environmental Research and Public Health	2017	More people live in cities than at any other time in human history and health inequities are increasing. Health inequities are avoidable differences in the social, environmental and political conditions that shape morbidity and mortality, and disproportionately burden the poor, racial, ethnic and religious minorities and migrants. By linking urban place and health inequities, research and action brings into sharp relief the challenges of achieving urban environmental justice. This article briefly reviews the complex definitions of urban places and how they can shape health equity in cities. I suggest that a more relational or integrated approach to defining urban places and acting on health equity can complement other approaches and improve the ability of public health to meet 21st century challenges. I close with suggestions for research and practice that might focus environmental public health on healthy urban place making. The practices include community driven map making, Health in All Policies (HiAP), promoting urban ecosystem services for health, and participatory and integrated approaches to urban slum upgrading. I conclude that if the global community is serious about the sustainable development goals (SDGs), greater attention must be paid to understanding and acting to improve urban places, living conditions and the social and economic conditions that can promote health equity.			PubMed_S 3
Sustainable Urban Transport Project (SUTP). https://www.sutp.org (GIZ - ASEM)	N		Web resource	rapid transit; busses; public transport; gender; active travel;	GIZ ASEM / ITDP	n/a	The Sustainable Urban Transport Project assists developing transport expertise in cities around the world to achieve their sustainable transport goals. They develop and disseminate resources on all aspects of sustainable urban mobility here. The SUTP Sourcebooks investigate the key areas important for a sustainable transport policy framework in developing cities. The resource material and a sourcebook for developing cities - see https://www.itdp.org/2003/12/01/sustainable-transport-a-sourcebook-for-developing-cities			peer

Record built from citation database, snowball or peer recommendation.	In sourcebook?	Sourcebook Ref	Type of resource	Key entry point(s)	Author or Publisher	Date	Description	Audience (text as in sourcebook, otherwise blank)	Good for these situations (text as in sourcebook, otherwise blank)	Search Id
India's peri-urban frontier: rural-urban transformations and food security. Fiona Marshall, Pritpal Randhawa. 2017. IIED. Book/Report, 40 pages	N		Briefing	peri-urban; food; nutrition; urban agriculture ;	IIED	2017	In this working paper, we examine rural-urban transformations in India in relation to changes in food production, access, consumption, nutritional quality and safety. We demonstrate how efforts to address malnutrition in India are decoupled from urban development initiatives and associated areas of policy and planning. We discuss how a more holistic, food security-based perspective, along with measures to support fragile peri-urban ecosystems and communities engaged with agriculture, could underpin processes to improve the health and nutrition of urban and peri-urban residents.			IIED
Healthy Built Environments: A review of the literature: Fact Sheets, UNSW, 2013. https://cityfutures.be.unsw.edu.au/research/city-wellbeing/city-wellbeing-resources/fact-sheets/	N		Evidence	planning policy; cities; neighbourhoods;	UNSW Australia. City Futures Research Centre.	2013	The Healthy Built Environments Program prepared a set of five fact sheets that summarise key points from a literature review. These are a set of briefings that show how to move from the evidence to potential planning policy statements.<br>Fact Sheet 1: A guide to "Healthy Built Environments: A Review of the Literature"<br>Fact Sheet 2: The Built Environment and Physical Activity<br>Fact Sheet 3: The Built Environment and Connecting and Strengthening Communities<br>Fact Sheet 4: The Built Environment and Providing Healthy Food Options<br>Fact Sheet 5: The Importance of Green Open Space			snowball
Assembly: Civic Design Guidelines: Promoting Civic Life Through Public Space Design. Center for Active Design. 2018. https://centerforactivedesign.org/assembly	N		Design guide	public speces; social capital;	Center for Active Design	2018	A design guide for creating well-designed and well-maintained public spaces as a force for building trust and healing divisions in local communities. The Assembly Guidelines capture the culmination of four years of research and collaboration—with input from 200+ studies, 50+ cities, and dozens of expert advisors—to provide evidence-based design and maintenance strategies for creating cities where people trust each other, have confidence in local institutions, and actively work together to address local priorities.			snowball

Record built from citation database, snowball or peer recommendation.	In sourcebook?	Sourcebook Ref	Type of resource	Key entry point(s)	Author or Publisher	Date	Description	Audience (text as in sourcebook, otherwise blank)	Good for these situations (text as in sourcebook, otherwise blank)	Search Id
INHERIT (INter-sectoral Health and Environment Research for InnovaTion). https://inherit.eu	N		Web resource	health equity; research; cities; HiAP; wider determinants;		2019	European project identifying ways of living, moving and consuming that protect the environment and promote health and health equity. INHERIT focuses on living (green space and energy efficient housing), moving (active transport) and consuming (food consumption and production). Encouraging modification of lifestyles, characterised by a 'take, make, consume, dispose' model of growth. INHERIT formulated scenarios for more sustainable future and designed, implemented and tested intersectoral initiatives to achieve triple-win. A booklet brings together results, relevant to professionals across health, environment, food, education, energy, transport, etc.; also policymakers at EU, national, regional and local level, and individuals to take action. Other resources are available from the website such as an extensive case study database.<br><br>The INHERIT Model includes (1) an integrated conceptual framework, including an analytical model, a governance model and an action and evaluation model (2) a set of qualitative and quantitative indicators and (3) a set of health, environment and social impact assessment tools and cost-effectiveness methods.			snowball
